# EREG-driven oncogenesis of Head and Neck Squamous Cell Carcinoma exhibits higher sensitivity to Erlotinib therapy

**DOI:** 10.7150/thno.47176

**Published:** 2020-08-25

**Authors:** Shuli Liu, Yang Wang, Yong Han, Weiya Xia, Ling Zhang, Shengming Xu, Houyu Ju, Xiangkai Zhang, Guoxin Ren, Liu Liu, Weimin Ye, Zhiyuan Zhang, Jingzhou Hu

**Affiliations:** 1 Department of Oral and Maxillofacial-Head and Neck Oncology, Shanghai Ninth People's Hospital, School of Medicine, Shanghai Jiao Tong University, Shanghai, China.; 2 Laboratory of Oral Microbiota and Systemic Diseases, Shanghai Ninth People's Hospital, College of Stomatology, School of Medicine, Shanghai Jiao Tong University, Shanghai, China.; 3 National Clinical Research Center for Oral Diseases, Shanghai Key Laboratory of Stomatology & Shanghai Research Institute of Stomatology, Shanghai, China.; 4 Department of Molecular and Cellular Oncology, The University of Texas MD Anderson Cancer Center, Houston, TX, 77030, USA.

**Keywords:** Epiregulin, HNSCC, EGFR, C-Myc, Erlotinib

## Abstract

**Rationale:** The oncogenesis of head and neck squamous cell carcinoma (HNSCC) is believed to result from oncogene activation and tumor suppressor inactivation. Here, we identified a new oncogenic role for the EREG gene in HNSCC.

**Methods:** The TCGA database and immunohistochemistry assay were used to analyze expression of EREG in HNSCC tissues. Immunoblotting was performed to identify the EGFR-mediated pathways altered by EREG. The role of EREG in oncogenesis was investigated in *vivo* and in *vitro*.

**Results:** Upregulated EREG expression predicted a poor prognosis and triggered HNSCC oncogenic transformation by activating the epidermal growth factor receptor (EGFR) signaling pathway. We also demonstrated the direct association of EREG with EGFR and that this binding required EGFR domains I and III and the N57 residue of EREG. Moreover, EREG overexpression was shown to promote HNSCC oncogenesis by inducing C-Myc expression, and the pharmacological inhibition of C-Myc rescued EREG-promoted HNSCC oncogenesis. Unlike other EGFR ligands, EREG could mimic EGFR mutations by sustaining the activation of the EGFR-Erk pathway, and high EREG expression was positively associated with the response to treatment with the EGFR inhibitor erlotinib. Furthermore, knockdown of EREG decreased sensitivity to erlotinib treatment *in vitro* and *in vivo*.

**Conclusions:** These results identify the EREG-EGFR-C-Myc pathway as a crucial axis that drives HNSCC oncogenesis and show that EREG expression could be a predictive functional marker of sensitivity to erlotinib therapy in HNSCC.

## Introduction

Head and neck cancer (HNC) represents the sixth most common cancer worldwide, and more than 90% of HNC cases are squamous cell carcinomas (SCCs) 1. Although the molecular basis for HNSCC is complicated, the epidermal growth factor receptor (EGFR) is known to play a vital role in HNSCC carcinogenesis and tumor progression 2. Increased EGFR activity leads to the activation of downstream signaling cascades, such as the PI3K/PTEN/AKT, ERK and JAK/STAT pathways, which promote cell proliferation, invasion and metastasis. Moreover, elevated protein expression of EGFR is a prognostic marker for poor survival in HNSCC patients. In addition, the approved EGFR antagonist(*i.e.*, the monoclonal antibody cetuximab) is the only validated molecular targeted treatment for HNSCC 3. However, cetuximab is expensive, requires weekly intravenous administration and carries the risk of allergic reactions. In contrast, EGFR tyrosine kinase inhibitors (TKIs; *e.g.*, erlotinib) are orally administered and have been widely used to treat both lung and pancreatic cancers. It is noteworthy that the EGFR-activating mutations identified in lung cancers have been well demonstrated to predict response to erlotinib. Lung cancers with EGFR mutations are often “driven” by EGFR activation and are sensitive to erlotinib, resulting in a prolonged lifespan for these lung cancer patients 2. However, EGFR mutations are infrequent in HNSCC, and erlotinib treatment shows marginal benefit in general in the absence of predictive biomarkers 4. Thus, the identification of predictive biomarkers for HNSCC could benefit a subpopulation of patients who may be more likely to respond to erlotinib treatment.

Epiregulin (encoded by the EREG gene) is a 46-amino acid protein that belongs to the epidermal growth factor (EGF) family. Epiregulin can bind to EGFR (ErbB1) and ErbB4 (HER4) and stimulate ErbB2 (HER2/Neu) and ErbB3 (HER3) signaling through ligand-induced heterodimerization 5, 6. Of interest, several mouse studies have shown that EREG deficiency resulted in reduced lung tumor promotion, and EREG overexpression fueled an oncogenic feedback loop that activated signaling pathways downstream of EGFR/ErbB4, suggesting that EREG might be a therapeutic target in non-small-cell lung carcinoma (NSCLC) 7,8. More recently, an elevated level of epiregulin was found to be associated with a greater benefit from cetuximab therapy, demonstrating the predictive role of EREG expression in the context of anti-EGFR therapeutic efficacy in metastatic colorectal cancer. In addition, it was previously reported that an EGFR blockade inhibited EREG expression and that EREG knockdown decreased clonogenic survival in basal-like HNSCC 9. Accordingly, we hypothesized that EREG expression would be a predictive functional marker of sensitivity to anti-EGFR TKIs in HNSCC.

## Materials and methods

### Cell Cultures, Plasmids and Reagents

The HNSCC-derived cancer cell lines HN30, HN4, HN6, HN12 and HN13 were kindly provided by the University of Maryland, School of Dentistry. The HEK293, CAL27 and FaDu cell lines were purchased from American Type Culture Collection (ATCC). Human oral keratinocytes (HOKs) were purchased from Chinese Beijing North Biotech, and the cell line was previously described 10. The human salivary adenoid cystic carcinoma (SACC) cell line SACC-83 was obtained from Peking University School of Stomatology. HN4, HN6, HN12, HN13, HN30, HEK293, and CAL27 cells were cultured in Dulbecco's modified Eagle's medium (DMEM; Gibco, NY, USA) supplemented with 10% fetal bovine serum (FBS), 1% glutamine, and 1% penicillin-streptomycin. SACC-83 and FaDu cells were cultured in RPMI-1640 (Gibco, NY, USA) with 10% FBS. All cells were maintained in a humidified atmosphere with 5% CO_2_ at 37°C.

EREG shRNA expression plasmids were purchased from Shanghai Era Biotech (Shanghai, China). Deletion mutants of EGFR plasmids were constructed as described previously 11. Human EREG was amplified from a HeLa cDNA library and subcloned into the vector pcDNA3.0, and we also generated EREG NQ mutants (N47Q, N57Q and N90Q) by performing site-directed mutagenesis. All sequences were verified by DNA sequencing. Recombinant human epiregulin, EGF, AREG, and TGF-α were purchased from R&D Systems (MN, USA). An anti-GAPDH antibody was obtained from Santa Cruz Biotechnology (CA, USA). Antibodies against EREG, EGFR, p-EGFR, ERK, p-ERK, AKT, p-AKT, ErbB2, p-ErbB2, ErbB3, p-ErbB3, STAT3, p-SATAT3, C-Myc, Axl and p-Axl were purchased from Cell Signaling Technology (MA, USA). HRP-conjugated secondary antibodies were purchased from Santa Cruz Biotechnology (CA, USA).

### Western Blotting and Immunoprecipitation (IP)

The experimental protocols for Western blotting and IP were performed as described previously 12. Briefly, RIPA lysis buffer was used to lyse the cells on ice, after which IP was performed with 2 µg of antibodies against EREG, EGFR or normal IgG (as a negative control) in 1.0 mg of whole-cell lysate. Then, cell lysates or immunoprecipitated cellular proteins were separated by SDS-PAGE in an acrylamide gel and transferred to a nitrocellulose membrane for further immunoblotting.

### Colony-Formation Assay

For the colony-formation assay, 1000 cells were seeded in 6-cm dishes (Corning, NY, USA) in triplicate per dish. The cells receiving various treatments were cultured for another 1-2 weeks in a humidified incubator at 37°C. The resulting colonies were fixed with 4% paraformaldehyde, stained with 0.5% crystal violet (Sigma, MO, USA) and counted.

### Three-Dimensional (3D) Culture

Three-dimensional (3D) cancer models were generated by seeding 10^4^ cells/well in ultra-low attachment (ULA) 96-well round-bottom microplates (Corning, Tewksbury, MA, USA). Multicellular cancer spheroids were obtained after the aggregation and compact clumping of cells. The spheroids were cultured for twelve days under standard culture conditions.

### Microarray Analysis

Expression profiling analysis was performed on HN4 and HOK cells using Affymetrix U133A microchips. Total RNA (20 µg) was transcribed to first-strand complementary DNA (cDNA) using SuperScript II reverse transcriptase (Invitrogen) with an oligo-dT primer that has a T7 RNA polymerase site on the 5′ end, and subsequent second-strand synthesis was performed to obtain double-strand cDNA. Then, the cDNAs were used in an in vitro transcription reaction in the presence of biotinylated nucleotides to generate single-stranded RNAs as recommended by Affymetrix. The biotin-labeled RNAs were fragmented and used for hybridization to Affymetrix human U133 gene chips. The data were analyzed using Affymetrix GeneChip software. In total, we found that 1240 genes met the criteria, of which 634 genes were upregulated, and 606 were downregulated in HN4 versus HOK cells.

### TCGA data mining

We performed data mining using the publicly available TCGA database (http://www.cbioportal.org/) to explore EREG gene mutation, deletion, and amplification levels in different cancers including HNSCC and the correlation between EREG mRNA and MYC mRNA in four different cancer tissues.

### Transfection of siRNAs

Specific siRNAs were used to knockdown EREG and EGFR expression in different cancer cells; a nontargeted siRNA was used as a negative control. EREG, EGFR and negative control siRNAs were chemically synthesized by Shanghai GenePharma Co. (Shanghai, China). According to the manufacturer's instructions, cells were transfected with 100 nM siRNAs diluted in Opti-Eagle's minimal essential medium (Invitrogen, CA, USA) using Lipofectamine 2000 (Invitrogen, CA, USA). After transfection, the cells were exposed to different treatments and lysed with RIPA lysis buffer for further analysis.

The sequences of siRNAs against the EREG sequence were as follows: siRNA-EREG (#1)-F: 5'-GCUCAAGUGUCAAUAACAAdTdT-3' and R: 5'-UUGUUAUUGACACUUGAGCdTdT-3'; (#2)-F: 5'-CCACCAACCUUUAAGCAAAdTdT-3' and R: 5'-UUUGCUUAAAGGUUGGUGGdTdT-3'; and (#3)-F: 5'-CUUUGACCGUGAUUCUUAUdTdT-3' and R: 5'-AUAAGAAUCACGGUCAAAGdTdT-3'. The siRNAs against the EGFR sequence were as follows: siRNA-EGFR(#1)-F: 5'-GUCGCUAUCAAGGAAUUAAdTdT-3' and R: 5'-UUAAUUCCUUGAUAGCGACdTdT-3'; (#2)-F: 5'-GGCUUGCAUUGAUAGAAAUdTdT-3' and R: 5'-AUUUCUAUCAAUGCAAGCCdTdT-3'; and (#3)-F: 5'-GUCCGCAAGUGUAAGAAGUdTdT-3' and R: 5'-ACUUCUUACACUUGCGGACdTdT-3'.

### Immunofluorescence

The immunofluorescence protocol was performed as described previously 13. Cultured cancer cells were rinsed with PBS three times, fixed with 3.7% formaldehyde, and then permeabilized with 0.1% Triton X-100. Subsequently, after 1 hr of blocking with 1% BSA, the cells were incubated with the primary antibody overnight. Then, the cells were washed and incubated with Alexa Fluor 488 (in the dark) or 594 donkey anti-rabbit IgG antibody (Invitrogen, NY, USA) for 1 hr at room temperature. The cells were then washed with PBS (containing 0.02% Tween 20) and stained by mounting onto a slide with aqueous mounting medium containing 0.5 mg/ml 4',6'-diamidino-2-phenylindole and examined using a fluorescence microscope.

### Reverse Transcription-Polymerase Chain Reaction (RT-PCR)

RT-PCR was performed as described previously 14. Total RNA samples were extracted with TRIzol Reagent (Invitrogen, CA, USA), after which cDNA was prepared from 1 mg of total RNA using a PrimeScript^TM^ RT Reagent kit (TaKaRa, Kyoto, Japan). Subsequently, the mRNA levels were determined by RT-PCR using the following primers: EREG (F: 5'-ATCCTGGCATGTGCTAGGGT-3' and R: 5'-GTGCTCCAGAGGTCAGCCAT-3'); C-Myc (F: 5'-ATGCCCCTCAACGTTAGC-3' and R: 5'-AGCTCGCTCTGCTGCTGC-3'); and GAPDH (F: 5'-TCCACCACCCTGTTGCTGTA-3' and R: 5'-ACCACAGTCCATGCCATCAC-3').

### Primary HNSCC Samples

Formalin-fixed, paraffin-embedded (FFPE) material was obtained from surgically resected HNSCC specimens from the Ninth People's Hospital (Shanghai, China). Eighty primary HNSCC patients were enrolled in this study and had not undergone prior radiotherapy or chemotherapy treatment. The average age of the patients was 57.35 years, ranging from 25 to 83 years. For each neoplastic tissue, histopathological diagnosis was performed according to the criteria of the World Health Organization. The TNM classification of the International Union against Cancer was used to determine the clinicopathological staging. Our study was approved by the Ethics Committee of Shanghai Ninth People's Hospital, Shanghai Jiao Tong University School of Medicine. We performed our study according to the recommendations of the Declaration of Helsinki.

### Immunohistochemistry Staining and Evaluation

Four-micron-thick tissue sections were deparaffinized, rehydrated, and washed with PBS for immunohistochemical analysis. After antigen retrieval by 0.01 mol/L citrate buffer (pH 6; DakoCytomation, CA, USA), the slides were incubated in a steamer for 30 min. The endogenous activity of each sample was blocked in a 3% hydrogen peroxide/PBS, avidin/biotin solution (Invitrogen, CA, USA), after which the samples were incubated with a polyclonal goat anti-human EREG antibody (R&D Systems, MN, USA) overnight at 4°C. The slides were then rinsed and incubated with a biotinylated-conjugated antibody and were labeled with streptavidin using peroxidase from a streptavidin-biotin kit (DakoCytomation, CA, USA). The samples were then stained with a 3,3'-diaminobenzidine tetrahydrochloride solution and counterstained with Mayer's hematoxylin for 2 min. The percentage of positive cells and the staining intensity were multiplied to produce a EREG immunohistochemical staining score. These judgments were made by two independent pathologists, neither of whom had knowledge of patient information. The staining index was divided into low and high groups, where a score of 0-4 (negative to medium) was defined as low EREG expression, and a score of 6 to 9 (strong) was defined as high EREG expression. Significant differences in EREG expression were detected, using measures analysis of Wilcoxon signed-rank test, between adjacent normal tissues and HNSCCs.

### In Vivo Tumorigenesis Assay

The animal study was approved by the Animal Ethics Committee of Ninth People's Hospital. All animal procedures were performed according to guidelines approved by the Shanghai Jiao Tong University School of Medicine. For the *in vivo* study, female SPF BALB/c nude mice (4 weeks old) were purchased from the Shanghai Laboratory Animal Center (Shanghai, China). All procedures were approved by the Laboratory Animal Care and Use Committees of our hospital. The tumor xenograft model was established with HN4, HN6 and HN30 cells, which exhibit high or low EREG expression *in vitro*. The cells (5×10^6^ cells/100 μL of PBS) were subcutaneously inoculated into the flanks of nude mice, and the tumor sizes were monitored three times a week. To calculate the tumor volumes, the formula (A)(B2)π/6 was used, where A indicates the longest dimension of the tumor, and B is the dimension of the tumor perpendicular to A. For HN30 cells, the mice were randomly divided into two groups when the mean tumor volume reached 40 mm^3^: the no treatment group and the erlotinib treatment group. Erlotinib was administered via oral gavage 4 days a week for 2 weeks at 50 mg/kg. All mice were sacrificed at the end of the treatment. Two-way repeated measure analysis of variance (RM ANOVA) was used to analyze the differences in the tumor volume between the two groups.

### Statistics

The experiments were repeated at least 2 times, and the results are presented as the mean±SD or SEM as indicated. A 2-tailed Student's t-test was used for intergroup comparisons. A P value less than 0.05 was considered significant.

## Results

### Upregulated EREG Predicts a Poor Prognosis in HNSCC

**Figure 1:**
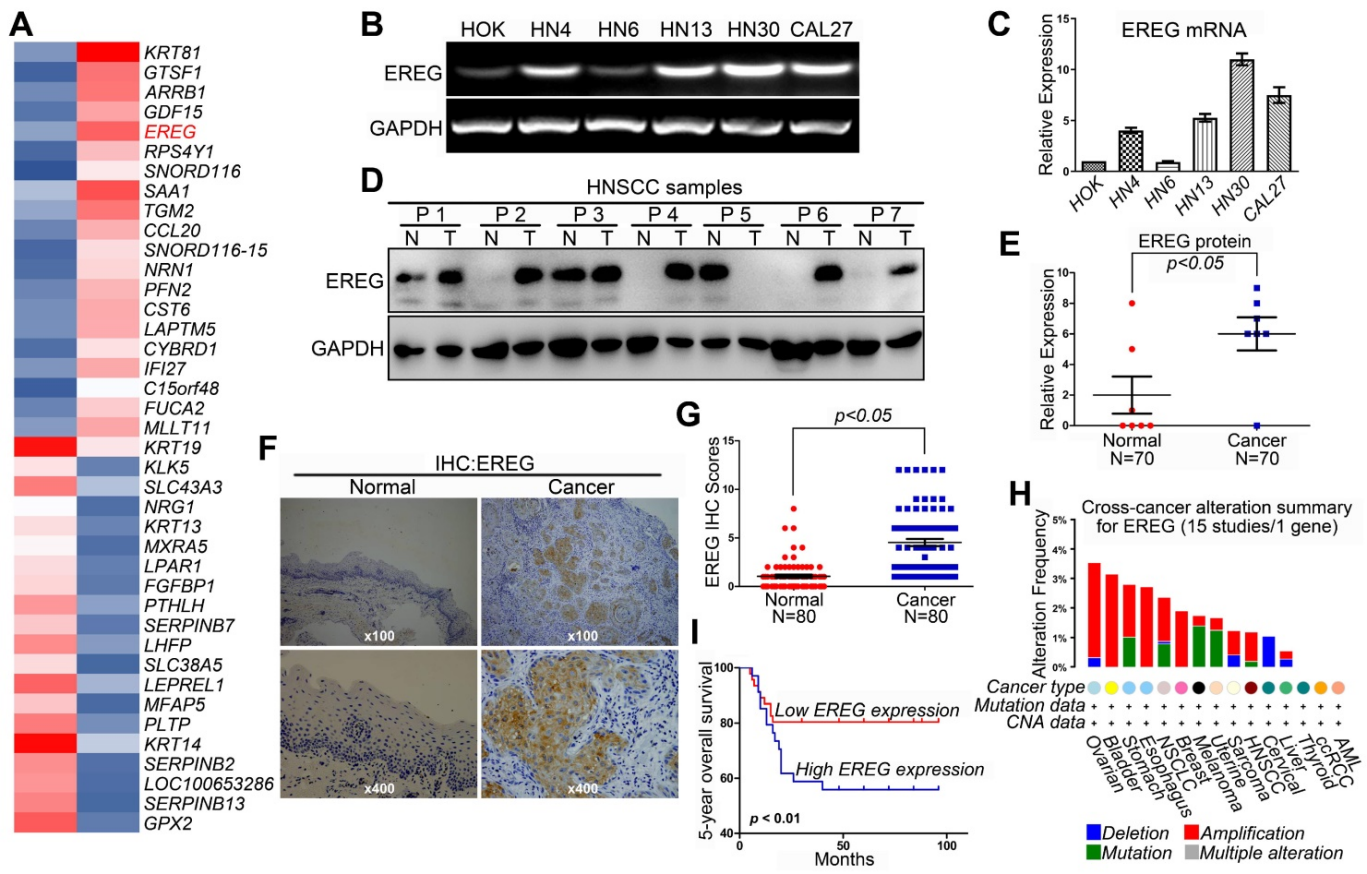
** High EREG expression predicts a poor prognosis in HNSCC patients. (A)** The microarray analysis between HN4 and HOK cells. **(B)** RT-PCR analysis of EREG mRNA levels in HOK and five other HNSCC cell lines. **(C)** Densitometric EREG mRNA data in B were normalized to GAPDH mRNA levels. **(D)** EREG expression from 7 paired cases of fresh-frozen HNSCC tumors was examined by Western blotting. **(E)** Densitometric EREG protein data in D were normalized to GAPDH protein levels. Significant differences were detected using a Wilcoxon signed-rank test (P < 0.005) in EREG expression between adjacent normal oral tissues and cancer tissues. **(F)** Representative images of EREG expression in normal tissues and HNSCC tissues via immunohistochemical (IHC) staining. **(G)** IHC scores of EREG expression in HNSCC tissues (n = 80) and paired adjacent normal tissues (n = 80). Significant differences were detected (P < 0.005) in EREG expression between adjacent normal tissues and HNSCC tissues. **(H)** EREG gene mutations in HNSCC tissues according to the cBioPortal for Cancer Genomics. **(I)** High EREG expression significantly correlates with the poor survival rate of HNSCC patients. The survival rates of patients with EREG-positive and EREG-negative tumors (P< 0.01) were determined using the Kaplan-Meier survival test

Revealing the molecular mechanisms underlying cancer progression and pathogenesis can lead to the discovery of new and effective strategies for early cancer detection and target therapy. Recent studies have indicated that high-throughput microarray technology is an efficient method for studying these processes 15-17. Thus, we first used a transcriptomic microarray analysis to select differentially expressed genes between the HNSCC cancer cell line HN4 and the human oral keratinocyte line HOK. We used a threshold of three-fold change for the differentially expressed genes obtained from the microarray. We found that a total of 1,240 genes met the criteria, of which 606 genes were upregulated, and 634 were downregulated in the cancer line compared with HOK cells (Table S1). Among these upregulated genes, EREG was one of the highest, and EREG expression in HN4 cells was increased by 32.9-fold compared with expression in HOK cells (Figure 1A). To confirm the microarray data, RT-PCR and Western blot analyses were performed to investigate the mRNA and protein levels of EREG in several HNSCC cell lines and normal HOK cells. As expected, both the mRNA and protein levels of EREG in the HNSCC cell lines were significantly higher than those in HOK cells (Figure 1B-C and Figure S1A). We also investigated the protein levels of EREG in 7 paired HNSCC specimens. Correspondingly, the protein levels of EREG were remarkably upregulated in 7 HNSCC tissues compared with those in the paired adjacent normal tissues (Figure 1D-E). These findings were further validated via immunohistochemistry (IHC) analysis of 80 paired primary HNSCC samples (Table 1). Notably, the EREG IHC score was significantly higher in cancer tissues than in normal tissues (Figure 1F-G). To further investigate EREG expression in HNSCC, we assessed the gene mutations, deletions, amplification and mRNA expression levels of EREG in a large cohort of HNSCC specimens provided by The Cancer Genome Atlas (TCGA). EREG gene mutations and amplifications were observed in a small proportion of patients (Figure 1H).

A Kaplan-Meier test was performed to further explore the relationship between EREG expression and the survival rate of HNSCC patients. The results showed that patients with high EREG expression had a significantly shorter overall survival rate than patients with low EREG expression (P< 0.01) (Figure 1I). By analysis of the overall survival based on EREG mRNA expression, we also found that patients with high EREG levels had a significantly shorter median overall survival than patients with low EREG levels in both HNSCC (Figure S1B-C) and other cancer types (Figure S1E-G). Although the protein expression levels of EREG and EGFR were not consistent in HNSCC (Figure S1A), the co-expression of EREG and EGFR also predicated a poor prognosis in HNSCC (Figure S1D). Taken together, these results demonstrate that upregulated EREG predicted poor prognosis in HNSCC.

### Epiregulin Promotes the Oncogenesis of HNSCC Both In Vitro and In Viv*o*

**Figure 2:**
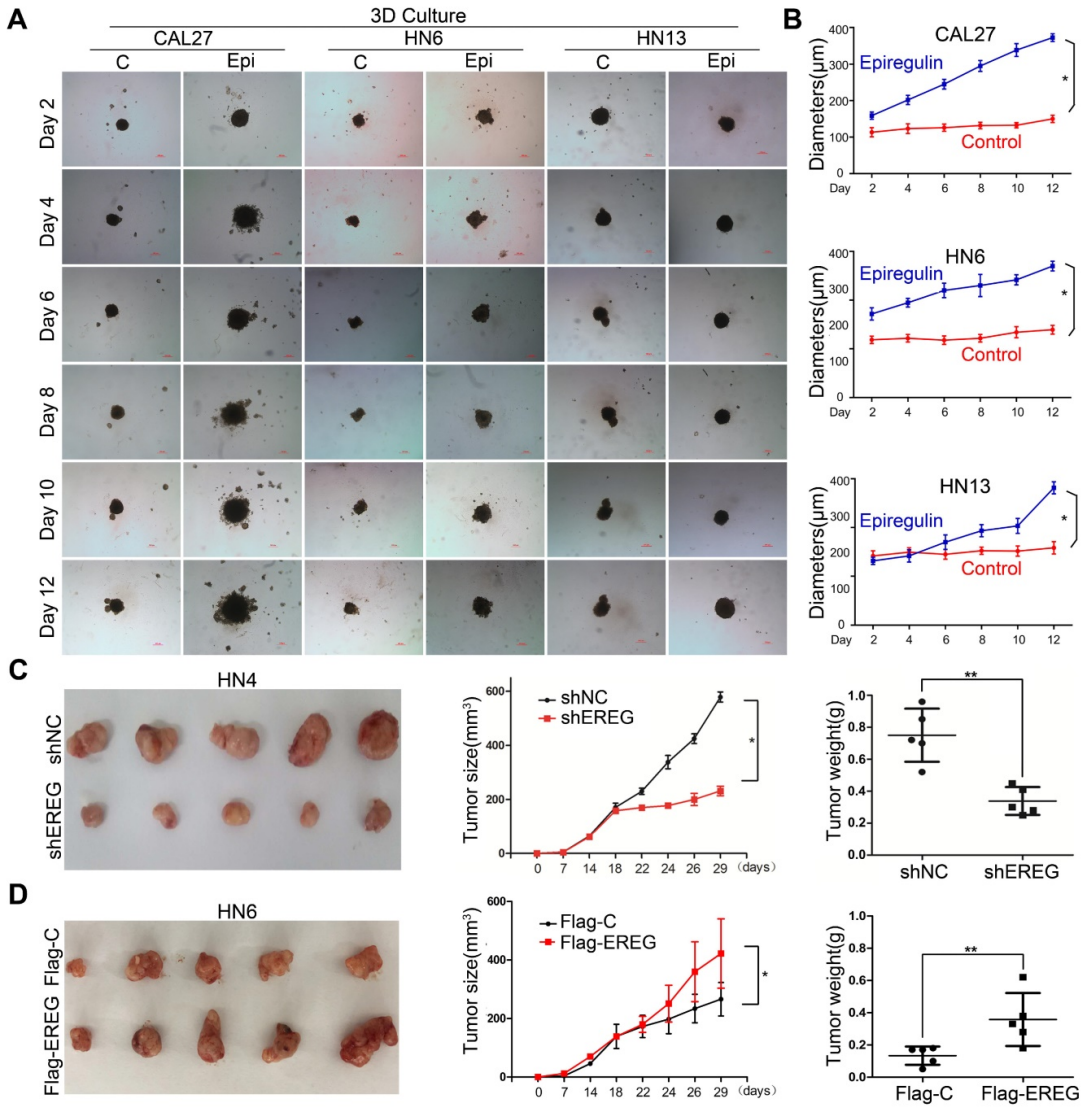
** EREG promotes HNSCC tumorigenicity *in vitro* and *in vivo*. (A)** Effect of EREG on the cell growth of 3D-cultured HNSCC cancer cell lines. Three HNSCC cell lines were seeded on day 0 and cultured in 3D conditions through day 12. Representative images of each cell line were captured on days 2, 4, 6, 8, 10 and 12. Each cell line was treated with or without EREG from day 1 through day 11. Scale bars indicate 100 µm.** (B)** The growth of 3D-cultured CAL27, HN6 and HN13 cells treated with or without EREG was analyzed. Each data point represents the mean value and standard deviation of 3 replicate wells. **(C)** HN4 cells stably transfected with control or EREG-specific shRNAs were injected into nude mice. Tumor growth was monitored every 3 days; tumor size and weight were recorded. The data are presented as the mean ± SEM from five mice. *P < 0.05 and **P < 0.01 for vector control cells compared with their EREG-knockdown clones.** (D)** HN6 cells stably transfected with control or Flag-EREG constructs were injected into nude mice. Tumor growth was monitored every 3 days, and tumor size and weight were recorded. The data are presented as the mean ± SEM from five mice. *P < 0.05 and **P < 0.01 for the vector control cells compared with the Flag-EREG clones.

To explore the role of EREG in oncogenesis in HNSCC, we exposed HNSCC cells to exogenous EREG (epiregulin). First, we examined the effects of epiregulin on colony-formation abilities in 2D cultures of HNSCC cells. Exogenous epiregulin treatment increased colony formation in both CAL27 and HN13 cells (Figure S2A-C), suggesting that EREG may promote the oncogenesis of HNSCC. Importantly, in a 3D culture system, which shows a better simulation of the *in vivo* tumor microenvironment, the spheroids was substantially larger for each of these cell lines treated with epiregulin than the control cells (Figure 2A-B). To further investigate the tumorigenic role of EREG *in vivo*, we analyzed whether the knockdown or exogenous expression of EREG affected *in vivo* tumorigenicity using a xenograft mouse model. As expected, the average size and weight of tumors in the EREG-knockdown group was significantly smaller than that observed in the control group (P< 0.05) (Figure 2C and Figure S2D). Moreover, the average tumor size and weight of in EREG overexpression group was significantly greater than that observed of the control group (P < 0.05) (Figure 2D and Figure S2E). Taken together, these results suggest that EREG expression in HNSCC cancer cells could affect their tumor supportive functions and plays a critical role in the oncogenesis of HNSCC, *in vitro* and *in vivo*.

### EREG Triggers EGFR Downstream Signaling

**Figure 3:**
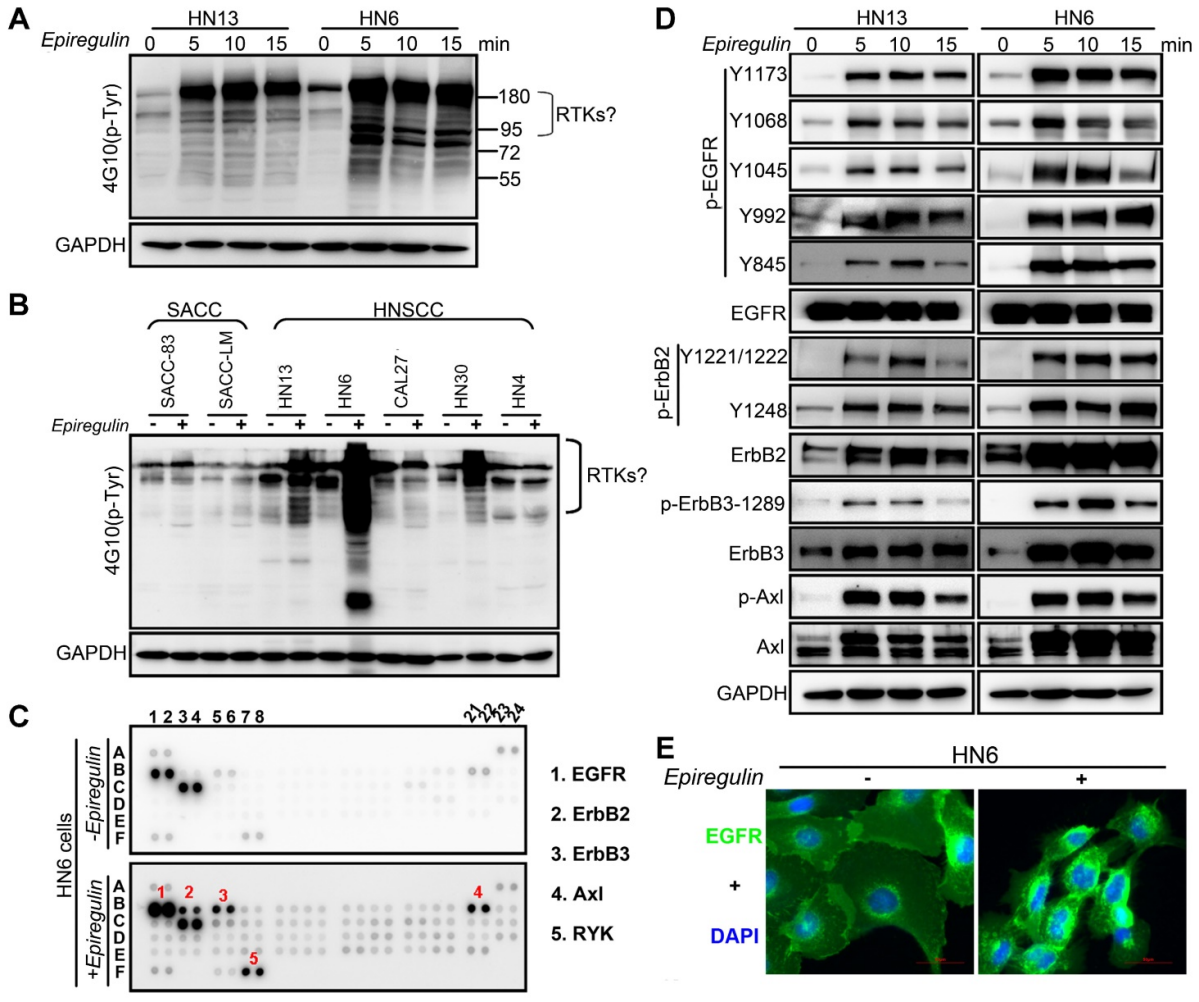
** EREG associates with EGFR and triggers EGFR signaling. (A)** Immunoblot (IB) of HN13 and HN6 cells treated with epiregulin (50 ng/ml) at the indicated time points and probed with an anti-phosphotyrosine (p-Tyr) antibody. **(B)** IB of SACC and HNSCC cancer cells treated with epiregulin (50 ng/ml) for 5 min and probed with an anti-p-Tyr antibody. **(C)** Human phospho-RTK antibody array analysis of HN6 cells serum starved for 24 hr, followed by epiregulin (50 ng/mL) treatment for 5 min. **(D)** IB of HN13 (left) and HN6 (right) cells treated with epiregulin (50 ng/ml) at various time points. **(E)** Immunofluorescence staining for EGFR in HN6 cells treated with or without 50 ng/mL epiregulin.

**Figure 4:**
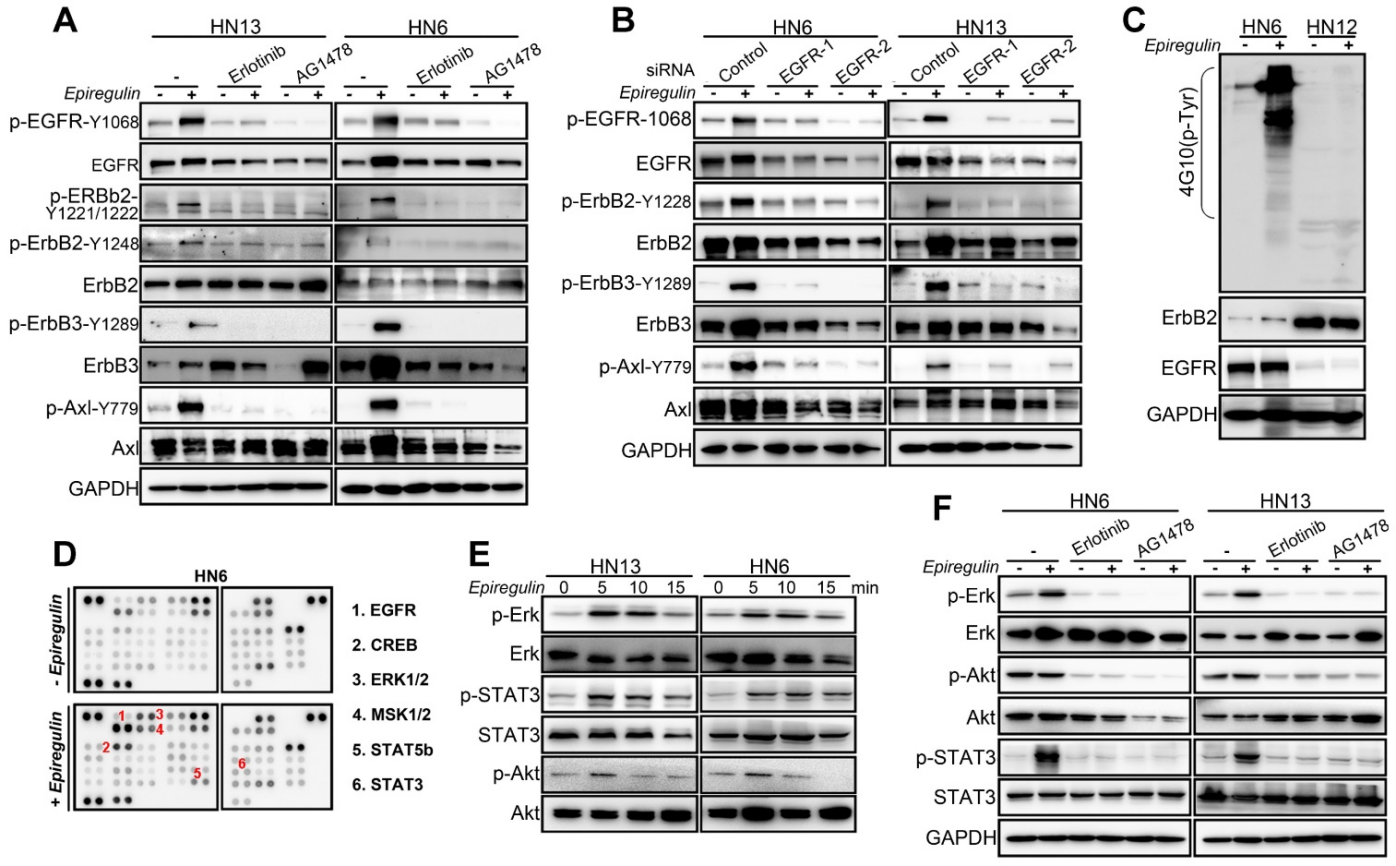
** EREG triggers EGFR downstream signaling in an EGFR kinase-dependent manner. (A)** HN13 and HN6 cells pretreated with erlotinib and AG1478 followed by epiregulin treatment and IB with the indicated antibodies. **(B)** IB of HN6 and HN13 cells transfected with individual small interfering RNAs (siRNAs) against EGFR in the presence or absence of epiregulin (50 ng/ml). **(C)** IB of HN6 and HN12 cells treated with or without epiregulin (50 ng/ml). **(D)** Human phosphokinase antibody array analysis of HN6 cells treated with or without epiregulin (50 ng/ml) for 5 min. **(E)** IB of HN13 (left) and HN6 (right) cells treated with epiregulin (50 ng/ml) at different time points. **(F)** HN6 and HN13 cells pretreated with erlotinib and AG1478 followed by epiregulin treatment and immunoblotting (IB) with the indicated antibodies.

To further explore how EREG induces oncogenic transformation, we investigated whether EREG triggers receptor phosphorylation and activation because EREG is a new member of the EGF family, and tyrosine phosphorylation is associated with HNSCC oncogenesis. Using the phosphotyrosine (p-Tyr) profile analysis, we found that EREG promoted signaling cascades of Tyr phosphorylation in multiple types of HNC cells. Importantly, the p-Tyr content that corresponded with a receptor tyrosine kinase (RTK)-equivalent molecular weight of 170-200 kDa was substantially increased by epiregulin treatment (Figure 3A, 3C and Figure S3A). We further performed an unbiased antibody array for human phospho-RTKs with or without epiregulin treatment in HN6 cells (Figure 3C). Different from previous reports that EREG binds directly to EGFR and HER4 to induce tyrosine phosphorylation (activation) of EGFR, HER2, HER3 and HER4, we found that epiregulin treatment increased the phosphorylation of EGFR, ErbB2, ErbB3 and Axl in HN13 and HN6 cells (Figure 3C-D). Using immunofluorescence microscopy, we detected internalized EGFR in the cytoplasm following treatment with epiregulin or transfection with an EREG plasmid (Figure 3E and Figure S3B). We also detected internalized ErbB2, ErbB3 and Axl in HN6 cells treated with epiregulin (Figure S3C). Moreover, among EGFR, ErbB2, ErbB3 and Axl, only EGFR was the direct target of EREG as knockdown EGFR or inhibited the kinase activity by inhibitors blocked phosphorylation of EGFR, ErbB2, ErbB3 and Axl (Figure 4A-B). Consistent with our finding, in HN12 cells, which express ErbB2 but not EGFR, did not respond to epiregulin stimulation (Figure 4C). Several well-recognized EGFR downstream molecules, such as ERK1/2 and STAT3, were also phosphorylated in response to EREG, as shown by the phospho-kinase array analysis (Figure 4D-E). Pretreating cells with EGFR-TKI, erlotinib, or AG1478 abolished ERK1/2, AKT and STAT3 activation (Figure 4F), indicating that EREG triggers EGFR downstream signaling in an EGFR kinase-dependent manner.

### EGFR Domains I and III and the N57 Residue of EREG are required for EREG-EGFR Interaction

**Figure 5:**
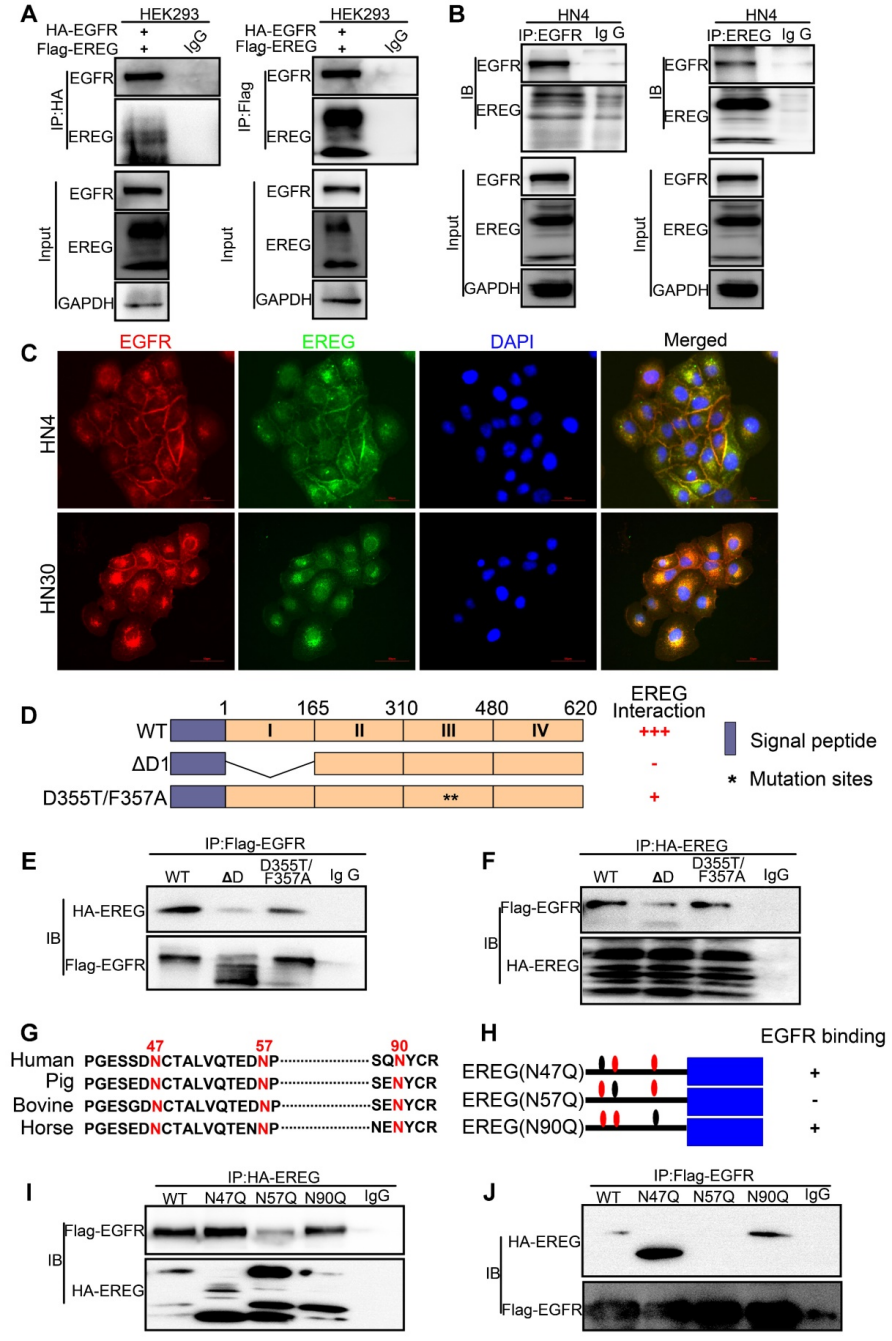
** EREG binds to EGFR via N57 and requires the EGFR domains I and III. (A)** HEK293 cells were transiently coexpressed with FLAG-EREG and HA-tagged EGFR. Cell extracts were immunoprecipitated separately with anti-FLAG or anti-HA antibodies, and the associated EGFR and EREG proteins were examined by Western blotting. **(B)** Endogenous EGFR and EREG were immunoprecipitated from HN4 cells, and bound endogenous EREG and EGFR were examined by Western blotting. **(C)** The cellular location of EGFR (red) and EREG (green) was examined by immunofluorescence staining (nuclei were stained with DAPI; blue). Scale bar, 50 μm. **(D)** Schematic diagram of the WT, domain I deletion (∆D1), and domain III mutation (D355T/F357A) constructs of EGFR (FLAG-EGFR-ECD). The numbers represent amino acid residues. **(E-F)** FLAG-tagged WT or deletion mutants of EGFR were coexpressed with HA-EREG in HEK293 cells. Extracts were immunoprecipitated with an anti-FLAG or anti-HA antibody, and bound EREG or EGFR was examined by Western blotting using the anti-HA or anti-FLAG antibody (for input control, see Figure **S4C**). **(G)** Sequence alignment of EREG from different species. **(H)** Schematic diagram of various EREG NQ mutants used in this study. The numbers indicate amino acid positions on the EREG. **(I-J)** HA-tagged WT or NQ mutants of EREG were coexpressed with FLAG-EGFR in HEK293 cells. EREG and EGFR were immunoprecipitated with anti-HA and anti-FLAG antibodies, respectively, and analyzed by Western blotting.

Previous studies and our above results confirm that EREG functional acts as an EGFR ligand. However, how EREG binds to EGFR to transduce signaling is still unclear. To further investigate the interaction of EREG with EGFR, we coexpressed FLAG-EREG and HA-EGFR in HEK293 cells and performed a co-IP experiment. After the immunoprecipitation (IP) of EGFR, we detected an associated EREG, and vice versa (Figure 5A). The IP of endogenous EREG and EGFR from HN4 and FaDu cells also demonstrated the presence of endogenous EGFR and EREG, respectively (Figure 5B and Figure S4A). Furthermore, using immunofluorescence microscopy, we detected the colocalization of EGFR and EREG in both HN4 and HN30 cells (yellow, merged images; Figure 5C and Figure S4B). Many EGFR ligands, such as EGF and ANG, interact with EGFR primarily through the extracellular domain (ECD) of EGFR. To further determine whether the domains I and III of the EGFR ECD, which are known to bind EGF, are also required for EREG binding, we generated a deletion construct of EGFR domain I (EGFR-∆D1; amino acids 1-165 deletion) and a critical EGF-binding EGFR domain III mutant, EGFR-D355T/F357A, and examined their abilities to bind EREG (Figure 5D). We found that the association of EGFR-∆D1 with EREG was significantly reduced compared with that of EGFR-wild-type (WT) with EREG. Compared with EGFR-WT, EGFR-D355T/F357A exhibited an 80% loss of binding to EREG (Figure 5E-F and Figure S4C-D). Together, these results suggested that the EGFR domains I and III are required for EREG binding and that the epitope of EREG binding to EGFR partially overlaps with the EGF-EGFR binding region.

N-glycosylation plays an important role in determining protein structure and function. In particular, membrane receptor protein glycosylation is important for protein-protein interactions, such as interactions between ligands and receptors, and affects protein activities 18. After initiation in the endoplasmic reticulum, protein N-glycosylation continues in the Golgi apparatus 19. Protein glycosylation is first catalyzed by a membrane-associated oligosaccharyltransferase complex that transfers a preformed glycan composed of oligosaccharides to an asparagine (Asn) side-chain acceptor located within the NXT (-Asn-X-Ser/Thr-) motif 20, 21. Therefore, we asked whether a glycosylation modification of EREG may play an important role in its binding to EGFR as a secretory protein. Thus, to identify the region of EREG required for EGFR binding, we performed a primary sequence alignment of EREG, searched for evolutionarily conserved NXT motifs in EREG amino acid sequences from different species (Figure 5G) and identified three conserved residues (N47, N57, and N90). Then, we mutated each of the three conserved asparagine (N) residues into glutamine (Q) residues; when the middle residue was mutated into alanine (N57Q), the association of this mutant with EGFR was inhibited (Figure 5H-J and Figure S4E-F), suggesting that the N57 residue may play a critical role in the binding of EREG to EGFR. The decreased binding of EREG-N57Q to EGFR was further validated *in vivo* by incubating HOK cells with conditioned medium (CM) containing secreted EREG-WT or EREG-N57Q mutant following transfection. As expected, in the EREG-N57Q group, the EGFR autophosphorylation level was decreased (Figure S4G). Together, these results suggested that the N57 residue of EREG is required for the efficient binding and activation of EGFR. A cocrystal structure needs to be further pursued to determine the molecular basis of the interaction between EREG-N57 and EGFR.

### EREG Induces C-Myc Expression in HNSCC

**Figure 6:**
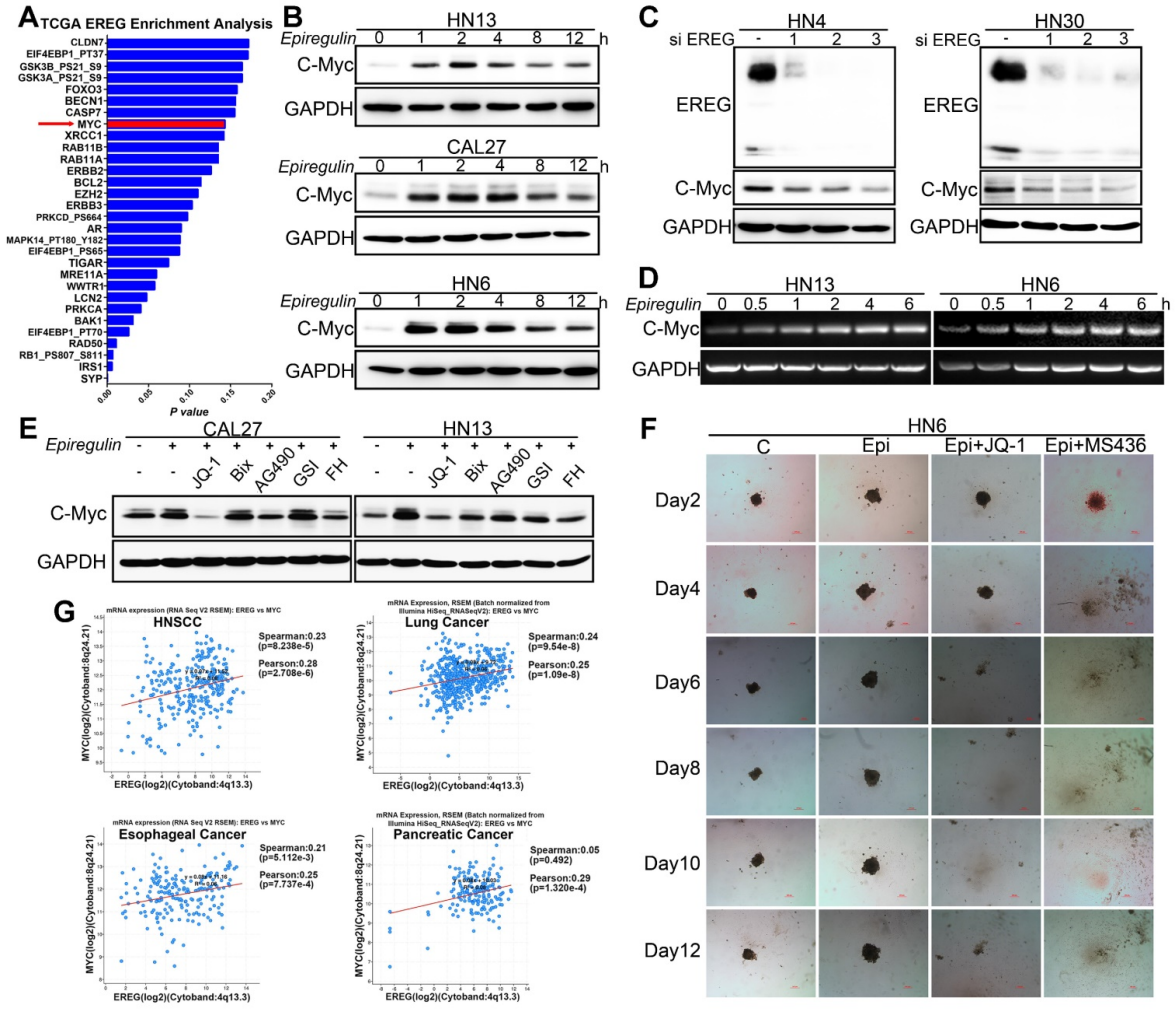
** EREG-induced C-Myc expression is required for EREG-promoted oncogenesis in HNSCC. (A)** EREG-related gene enrichment analysis from TCGA using the cBio Cancer Genomics Portal. **(B)** Western blot analysis for C-Myc from three different HNSCC cell lines treated with 50 ng/mL epiregulin as indicated. **(C)** Western blot analysis of EREG and C-Myc expression in HN4 and HN30 cells after transfection with siEREG or siNC siRNAs. **(D)** RT-PCR analysis of C-Myc mRNA levels in HN13 and HN6 cells treated with 50 ng/mL epiregulin as indicated. **(E)** CAL27 and HN13 cells were pretreated with various inhibitors for 1 h followed by stimulation with epiregulin for 2 h. The level of C-Myc was examined by Western blot analysis. **(F)** 3D culture of HN6 cells treated with or without epiregulin and BET BD inhibitors. Scale bar=100 μm. **(G)** A correlation was found between EREG and C-Myc at the mRNA level in four gene expression data sets.

To further investigate the potential target genes regulated by EREG, we performed EREG enrichment analysis from the TCGA cohort (TCGA, Nature 2015) by using the cBio Cancer Genomics Portal (http://cbioportal.org) 22. Among those genes, the MYC oncogene was one of the top EREG-regulated genes (Figure 6A) and codes for a transcription factor that is overexpressed in many human cancers. As previously reported, c-Myc mainly regulates the expression of genes involved in cell proliferation, differentiation, growth, apoptosis, and self-renewal, and overexpressed c-Myc has been causally linked to tumorigenesis 23-26. Thus, we believe that EREG may promote HNSCC oncogenesis by upregulating c-Myc expression. To confirm this possibility, we first investigated whether c-Myc was regulated by EREG in our system. Our results showed that rhEREG (epiregulin) treatment significantly increased C-Myc protein levels in three HNSCC cells (Figure 6B). The immunofluorescence results confirmed those findings, as the staining signal of c-Myc was dramatically increased in both HN6 and CAL27 cells following EREG treatment (Figure S5A). To further confirm this observation, we also knocked down EREG expression in both HN30 and HN4 cells. The knockdown efficiency of EREG by siRNA was approximately 90%, and knockdown of EREG dramatically downregulated c-Myc expression in both cell lines (Figure 6C), suggesting that c-Myc was regulated by EREG. As shown in Figure 6D, treatment of HN13 and HN6 cells with epiregulin also increased the level of c-Myc mRNA expression. Consistent with this notion, when we pretreated the cells with the transcription inhibitor actinomycin D, we found that c-Myc induction by EREG was completely blocked (Figure S5B). In addition, we incubated cells with epiregulin for 2 hr and then treated the cells with the protein translational inhibitor cycloheximide (CHX) (Figure S5D). We found that the rate of c-Myc degradation was similar to that in cells without epiregulin pretreatment (Figure S5E). Taken together, these data indicate that EREG induces c-Myc expression by promoting its transcription in HNSCC cells.

Recently, many studies have found that inhibition of bromodomain-containing protein 4 (BRD4) with pharmacological BET-specific bromodomain (BD) inhibitors can effectively block MYC expression in multiple myeloma, acute myeloid leukemia, and Burkitt's lymphoma 27-29. We thus hypothesized that BET inhibition may be related to EREG-mediated c-Myc transcriptional activation. As expected, when pretreated with three different BET inhibitors, we found that EREG-induced C-Myc expression was completely blocked in both CAL27 and HN13 cells (Figure 6E and Figure S5F-G). Moreover, in the high EREG expression cell lines HN4 and HN30, treatment with BET inhibitors also completely blocked C-Myc expression (Figure S5J). Next, we investigated whether BR inhibition also suppressed the EREG-promoted oncogenesis of HNSCC cells. We examined the effects of two BET inhibitors on the oncogenesis of HNSCC cells that were treated with or without EREG. EREG treatment significantly increased cell proliferation in our 3D-culture system assay. However, this effect was significantly inhibited when C-Myc was blocked by BET inhibitors in HN6 cells (Figure 6F and Figure S5I). These results demonstrate that BET BRs are crucial for EREG-mediated c-Myc expression.

**Figure 7:**
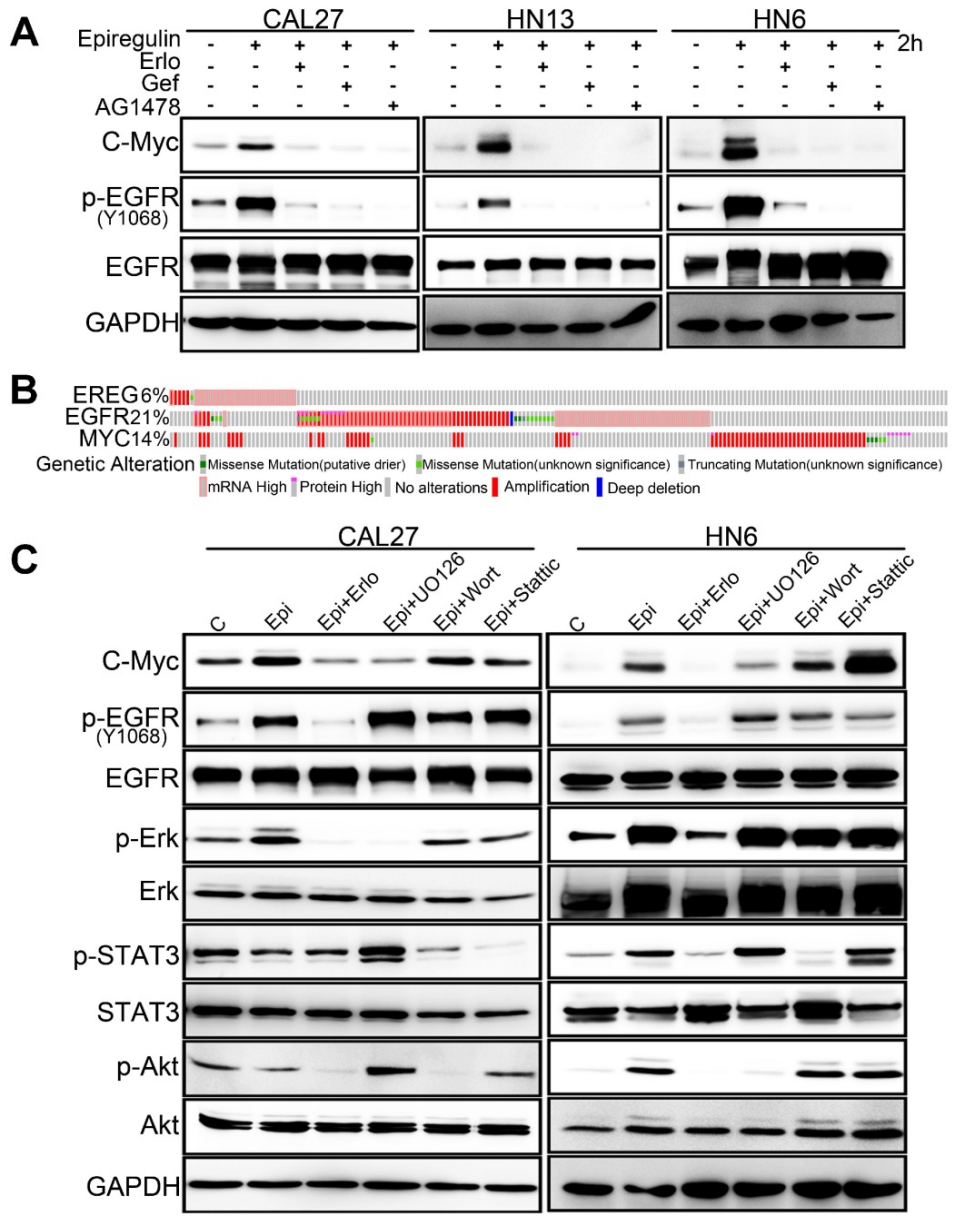
** EREG-induced C-Myc expression depends on EGFR activity. (A)** Western blot analysis of C-Myc, p-EGFR, and EGFR from tumor cell lines pretreated with various EGFR inhibitors for 1 h followed by stimulation with epiregulin for 2 h. **(B)** OncoPrint of EREG-EGFR-MYC pathway alterations in HNC. Genomic alterations of different members of this pathway are mutually exclusive. **(C)** Western blot analysis of C-Myc, p-EGFR, EGFR, p-AKT, AKT, p-ERK, ERK, p-STAT3, and STAT3 from CAL27 and HN6 cells pretreated with various inhibitors for 1 h followed by stimulation with epiregulin for 2 h.

EREG binds to EGFR and triggers its downstream signaling pathways. To further validate whether EGFR activation is required for EREG-induced C-Myc expression, we explored the effects of different EGFR inhibitors. Our results showed that all these inhibitors, including erlotinib, gefitinib and AG1478, completely blocked EREG-induced expression of C-Myc in all three cells (Figure 7A). To investigate which downstream signaling pathway of EGFR is required for EREG-induced expression of C-Myc, we explored the effects of ERK, AKT and STAT3 pathway inhibitors. The results showed that the ERK inhibitor UO126 completely blocked EREG-induced C-Myc expression, whereas the STAT3 and AKT inhibitors showed only partial effects (Figure 7C). Taken together, these results strongly suggest that EGFR activation is crucial for EREG-induced C-Myc expression and that ERK signaling plays a major role in mediating this effect.

To further examine the EREG-C-Myc relationship, we also analyzed the expression of EREG and C-Myc from four gene expression data sets comprising four different types of tumor samples (Figure 6G). We noticed a positive correlation in the mRNA expression level of EREG and C-Myc in these four gene expression data sets. To further search for genomic alterations in the EREG-EGFR-C-Myc pathway from the TCGA data, we performed analysis though the cBio Cancer Genomics Portal (http://cbioportal.org) as previously described 30. The OncoPrint analysis shows that alterations in genes from the EREG-EGFR-C-Myc pathway appear to be mutually exclusive (Figure 7B), suggesting that overexpression of these genes has a similar functional role.

### EREG Mimics the EGFR Mutation by Sustaining Activation of the EGFR-Erk Pathway in HNSCC

**Figure 8:**
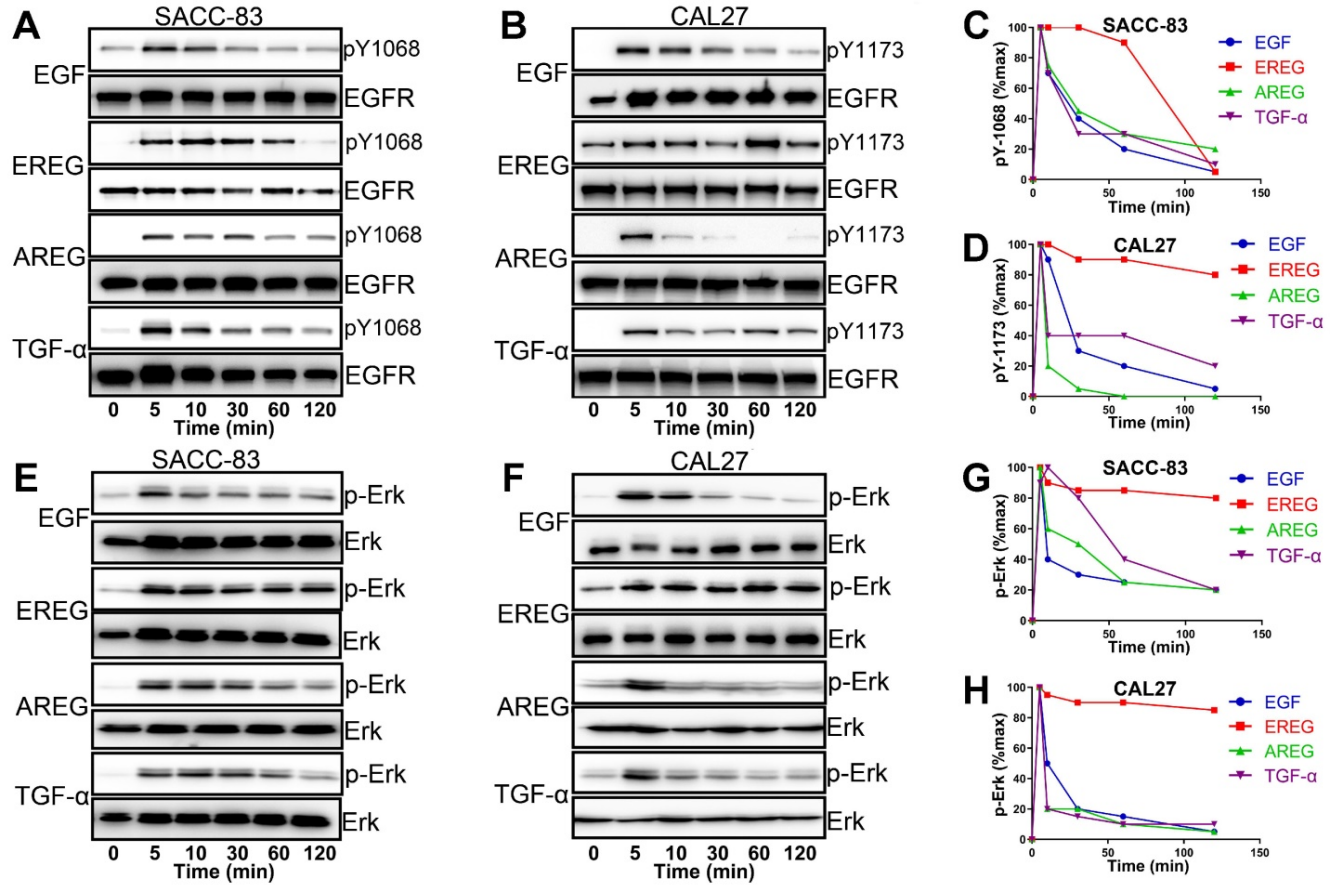
** EGFR-Erk activation by epiregulin is sustained. (A)** Representative time courses of EGFR phosphorylation at Y1086 in SACC-83 cells induced by EGF, EREG, AREG or TGF-α. An anti-EGFR antibody was used as a loading control. **(B)** Representative time courses of EGFR phosphorylation at Y1173 in CAL27 cells induced by EGF, EREG, AREG or TGF-α. An anti-EGFR antibody was used as a loading control. **(C-D)** Quantification of EGFR phosphorylation time courses, normalized by the signal at 5 min. The data are plotted on the same graph for multiple independent experiments quantitating phosphorylation at Y1068 and Y1173. **(E-F)** Representative time courses of Erk phosphorylation in SACC-83 and CAL27 cells induced by different EGFR ligands. **(G-H)** Quantification of Erk phosphorylation time courses, normalized by the signal at 5 min. The data are plotted on the same graph for multiple independent experiments quantifying Erk phosphorylation.

EGFR activation is induced by several different growth factors. Individual EGFR ligands also induce qualitatively and quantitatively different downstream signals that are linked to unique phenotypes. However, how different ligands could promote distinct cellular signaling responses through the same RTK remains unclear from the current mechanistic understanding 31-33. To understand the difference in EREG from other EGFR ligands in activating EGFR signaling, we first treated HNSCC cells with different EGFR ligands. As shown in Figure 8A and B, epiregulin-induced phosphorylation of EGFR was substantially more sustained than treatment with EGF, AREG or TGF-α. Whereas EGF-induced EGFR phosphorylation at Y1086 or Y1173 returns to baseline at approximately 30 mins of initial stimulation, it remains elevated even 60-120 min after initial stimulation with EREG (Figure 8C-D). Because Erk signaling plays a major role in mediating the EREG-EGFR-C-Myc pathway, it is important to determine whether EREG also sustained Erk signaling activation. As expected, Erk was substantially more sustained following activation with EREG than with EGF, AREG or TGF-α (Figure 8E-F). Similar to EREG-induced EGFR phosphorylation, EREG-induced Erk phosphorylation also remained elevated, even 120 min after initial stimulation with EREG (Figure 8G-H). These findings suggest that EREG can mimic EGFR mutations in HNSCC by sustaining the activation of the EGFR-Erk pathway and may have important biological functions that are different from those of other ligands.

### Increased Expression of EREG Predicts Higher Sensitivity to Erlotinib Treatment in HNSCC

**Figure 9:**
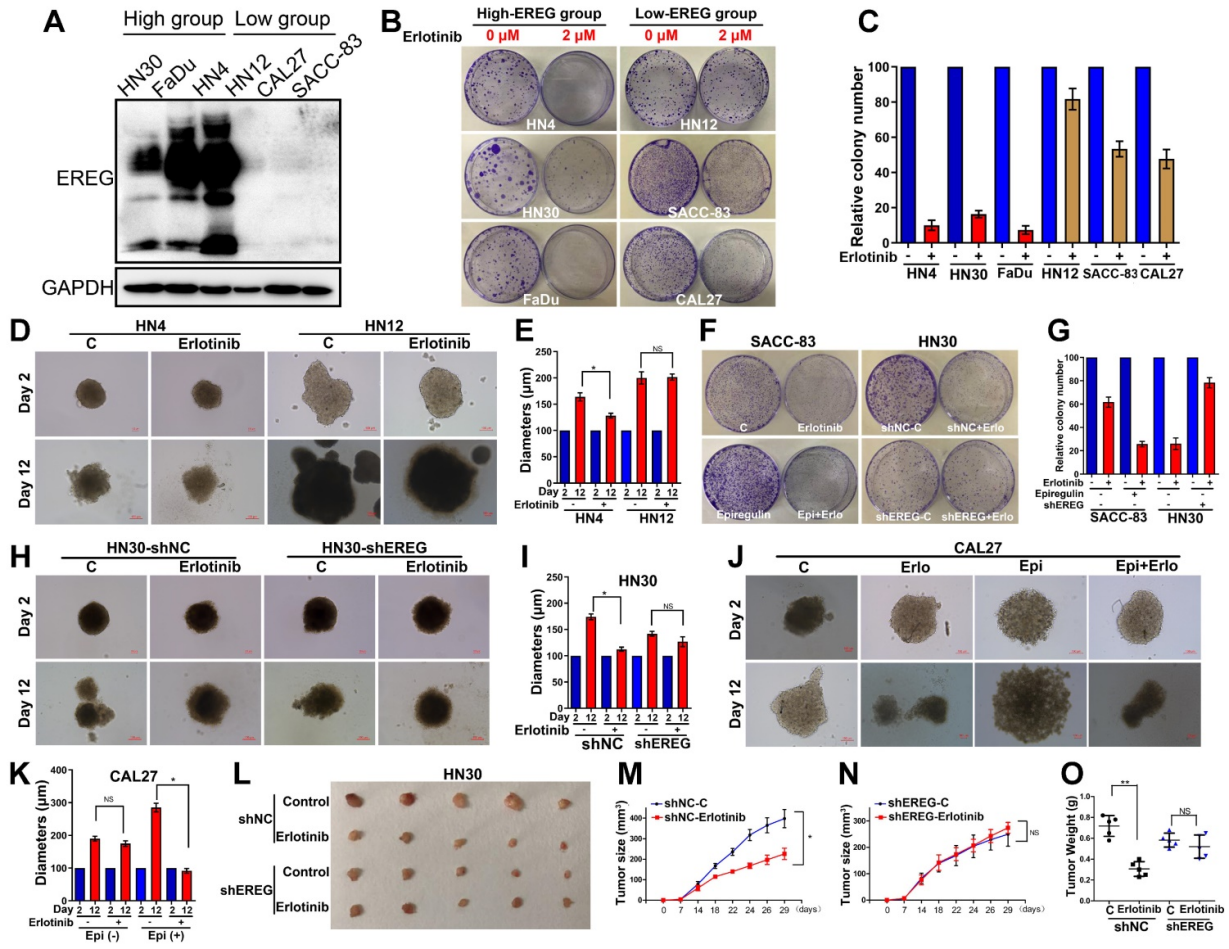
** High EREG expression predicted a better sensitivity to erlotinib treatment in HNSCC. (A)** Cell extracts were prepared from 6 HNC cell lines, which were divided into two groups based on EREG expression, and EREG expression was analyzed by Western blotting. **(B-C)** Colony formation was assessed in many cancer cell lines based on EREG expression levels with erlotinib (2 µM) treatment. The number of colonies was calculated. **(D-E)** Effect of erlotinib on the cell growth of 3D-cultured HN4 and HN12 cells. High-EREG-expressing HN4 and low-EREG-expressing HN12 cell lines were seeded on day 0 and cultured in 3D conditions through day 12. Representative images of each cell line were captured on days 2 and 12. Each cell line was treated with or without erlotinib from day 1 through day 11. Scale bars indicate 100 µm. **(F-G)** Colony-formation analysis was performed on SACC-83 and HN30 cells. The low-EREG-expressing SACC-83 cells were treated with erlotinib, epiregulin or erlotinib plus epiregulin, and the high-EREG-expressing HN30 cells stably transfected with control or EREG-specific shRNAs were treated with or without erlotinib. The number of colonies was calculated. **(H-I)** Effect of erlotinib on the cell growth of 3D-cultured high-EREG-expressing HN30 cells stably transfected with control or EREG-specific shRNAs. Scale bars indicate 100 µm. * P<0.05. **(J-K)** Effect of epiregulin and erlotinib on the cell growth of 3D-cultured CAL27 cells. Low-EREG-expressing CAL27 cell lines were seeded on day 0 and cultured in 3D conditions through day 12. Representative images of each cell line were captured on days 2 and 12. Each cell line was treated with epiregulin, erlotinib or epiregulin plus erlotinib from day 1 through day 11. Scale bars indicate 100 µm. **(L-O)** HN30 cells stably transfected with control or EREG-specific shRNAs were injected into nude mice followed by treatment with or without erlotinib. Tumor growth was monitored every 3 days; tumor size and weight were recorded. The data are presented as the mean ± SEM from five mice.

Similar to the EGFR-activating mutations in lung cancer patients, identifying HNSCCs that are sensitive to EGFR-TKI could be a key aspect of the EGFR-TKI treatment response. Our results demonstrating that EREG in HNSCC can mimic EGFR mutations by sustaining activation of the EGFR-Erk pathway raised the question of whether EREG could be used to identify the patient group most responsive to EGFR-targeted therapies, similar to EGFR-activating mutations in lung cancer. Thus, we first evaluated EREG expression in six HNSCC cell lines and divided them into high- or low-EREG-expression groups (Figure 9A). The IC50 of erlotinib in 6 HNSCC cell lines is shown in Supplementary Figure S6A. Notably, the growth inhibition due to erlotinib treatment of HN4, HN30 and FaDu cells that expressed high EREG protein levels was significantly higher than that of cells expressing lower levels of EREG (Figure 9B-C). Importantly, in the 3D culture system, the size inhibition of the spheroids by erlotinib was substantially larger for HN4 cells than for HN12 cells (Figure 9D-E). These results strongly demonstrated that higher levels of EREG rendered HNSCC more sensitive to erlotinib and that high EREG expression has the potential to serve as a biomarker to predict erlotinib response in HNSCC patients.

### Knockdown of EREG Decreases Sensitivity to Erlotinib Treatment

To further study whether loss of EREG was sufficient to impact erlotinib efficacy, we first examined the effects of exogenous EREG on erlotinib sensitivity in SACC-83 and CAL27 cells that express a relatively low level of EREG. Exogenous EREG significantly increased sensitivity to erlotinib treatment in both SACC-83 and CAL27 cells (Figure 9F-G and J-K). We also knocked down EREG expression in HN30 cells (Figure S6B). Knockdown of EREG decreased the sensitivity to erlotinib treatment and the size of 3D culture spheroids (Figure 9F-I). We further confirmed the *in vitro* results using a nude mouse xenograft model. Strikingly, the sizes and weights of tumors in mice injected with EREG-knockdown HN30 cells decreased more substantially than those injected with vector control cells (Figure 9L-O). These results suggest that efficient knockdown of EREG expression decreased sensitivity to erlotinib treatment *in vitro* and *in vivo.*

## Discussion

**Figure 10:**
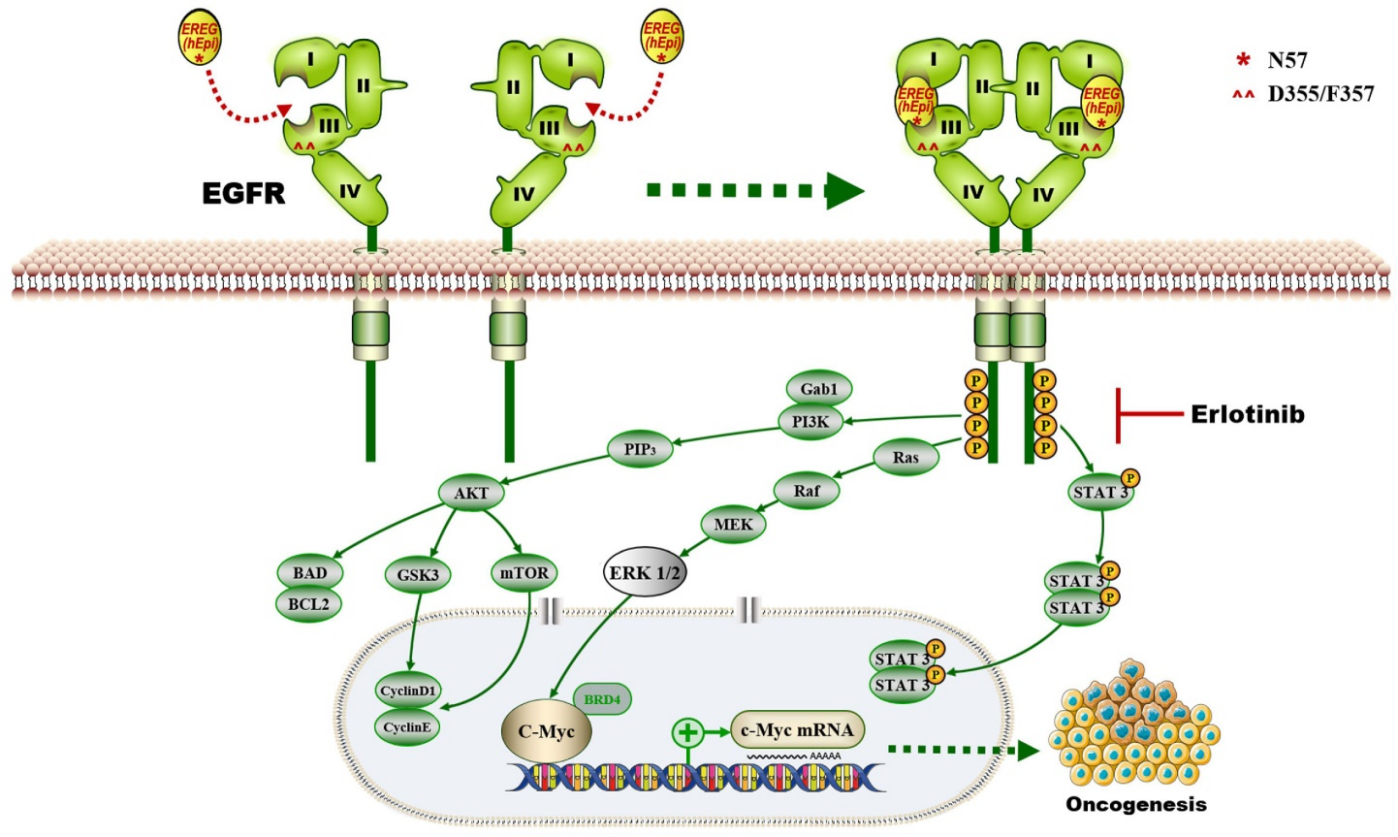
The proposed model showing that EGFR domains I and III and the N57 residue of EREG are required for EREG-induced EGFR-Erk-C-Myc signaling activation, which in turn promotes oncogenesis and increases erlotinib sensitivity in HNSCC patients.

In this study, we provide a model (Figure 10) showing that higher levels of epiregulin in the HNSCC microenvironment induce its binding to EGFR and activates the EGFR-Erk-C-Myc signaling axis, which in turn promotes tumorigenesis in HNSCC. In contrast to other members of the EGF family 34, EREG is mainly expressed in the placenta and in peripheral blood leucocytes. It was reported that overexpressed EREG promotes migration and invasion of oral cancer cells *in vitro*. The effects are likely mediated by the activation of ERK1/2, Akt, and Cox2 35. As previously reported, EREG activates not only homodimers of ErbB1 and ErbB4 but also all possible heterodimeric ErbB complexes 6. Thus, the biological functions of EREG may be distinct from other EGFR ligands. In our study, we found that EREG was remarkably upregulated in the HNSCC cancer cell line HN4 compared with HOK cells, as determined by microarray analysis. Furthermore, we examined the clinical significance of EREG by analyzing the correlation between EREG expression and clinical outcome in patients with HNSCC. Our data suggested that patients with increased expression of EREG in HNSCC had worse overall survival, consistent with findings described previously 36.

Although EREG upregulation has been found in many cancers, the molecular mechanism by which EREG promotes cancer progression in most cancers remains unclear. Previous studies have reported that OSCC patients with a high expression of epiregulin had a significantly lower survival rates than those with low expression 36. However, the contributions and molecular mechanism of EREG regarding HNSCC initiation and growth have not been investigated before this study. Here, we demonstrate that EREG contributes to the oncogenesis of HNSCC possibly through increased expression of the transcription factor C-Myc and ERK signaling, which plays a central role in mediating the effect. We identified that the EGFR domains I and III and the N57 residue of EREG are required for EREG-EGFR interaction. Moreover, EREG can mimic EGFR mutations in HNSCC by sustaining activation of the EGFR-Erk pathway. Our study also indicates that the EREG-EGFR-Erk-C-Myc axis represents a druggable target for treating HNSCC.

Aberrant EGFR expression is common in many epithelial tumors. Recently, many studies have focused on the development of candidate anticancer drugs that target EGFR 37. Somatic mutations in exons encoding the tyrosine kinase domain of EGFR lead to the constitutive activation of the kinase. Studies have found that lung adenocarcinomas sensitive to erlotinib often harbor EGFR mutations and that EGFR mutations are predictors of sensitivity to EGFR TKIs, increasing the treatment benefits; these results have recently been validated 38-41. Because HNSCC is known to progress rapidly, it is critical to identify any predictive biomarkers to increase treatment efficacy. However, the EGFR-sensitizing mutations observed in lung cancers are very uncommon in HNSCC, and there are currently no predictive markers for erlotinib response in clinical use. Markers that in some way reflect the general dependency of tumors on EGFR ligand signaling would be a promising alternative. It was initially reported that EREG expression and the efficacy of the EGFR inhibitors are closely correlated in colorectal cancer 42. Jonker et al also found that patients with higher EREG expression appear to benefit more from cetuximab therapy than those with low EREG expression in KRAS-WT colorectal cancer 43. The molecular mechanism for this association may be attributed to the stimulation of EGFR by EREG through a positive feedback autocrine loop 42. Other studies also found a correlation between sensitivity to cetuximab and gefitinib with the basic release of EGF, TGF-α and AREG in 10 NSCLC and 4 HNSCC cell lines 44. The authors identified AREG as a candidate marker for sensitivity to these drugs, and knockdown of AREG gene expression inhibited cancer cell growth.

Interestingly, a French group previously reported that the autocrine production of the EGFR ligand EREG could be a predictive functional marker of “basal-like” HNSCC sensitivity to EGFR blockade 9. Because basal-like HNSCC aberrantly expresses factors involved in EGFR signaling, including the upregulated expression of the EGFR ligand epiregulin, the authors proposed that the sensitivity of basal-like cells to EGFR blockade results from their addiction to an oncogenic autoamplifying loop characterized by high expression of epiregulin and amphiregulin. Moreover, EGFR blockade efficiently downregulated transcripts for the EGFR ligand epiregulin in basal-like HNSCC cells and reduced their clonogenic survival. Our results agree with their studies in that high EREG expression could be a predictive functional marker of sensitivity to EGFR blockade in basal-like HNSCC. Our study focused on investigating this possibility at the *in vitro* level, and we provide support for our proposal with *in vivo* results. More importantly, in our study, we observed that EREG induces sustained EGFR activation (rather than transient activation), leading to sustained Erk activation. This finding is important because the EGFR mutation is rare in HNSCC, and EREG likely contributes to the autoactivation of the EGFR pathway, which mimics the EGFR mutation. Thus, it is reasonable that EREG expression predicts increased sensitivity to erlotinib treatment in HNSCC. Additionally, EREG as a marker of EGFR-targeted therapy needs to be verified with clinical trials.

In summary, our study provided insights into the pathological relevance between EGFR and EREG in HNSCC. Moreover, we identified that EREG expression has the potential to serve as a biomarker to predict response to erlotinib treatment and to stratify HNSCC patients, particularly those with EREG^high^-EGFR^+^ disease.

## Supplementary Material

Supplementary figures.

Supplementary table.

## Figures and Tables

**Table 1:** The baseline characteristics of HNSCC patients include in the study

Characteristics	Patients	
	NO.	%
Age,years		
≤60	49	61.3
>60	31	38.7
Sex		
Male	39	48.8
Female	41	51.2
T-primary tumor size		
T1	22	27.5
T2	34	42.5
T3	14	17.5
T4	10	12.5
N-regional lymph node		
Negative	53	66.3
Positive	27	33.7
TNM stage		
I	23	28.75
II	23	28.75
III	18	22.5
IV	16	20.0
Histopathological type		
Grade 1	35	43.75
Grade 2	36	45.0
Grade 3	9	11.25
Smoking history		
Yes	27	33.8
No	53	66.2
Alcohol history		
Yes	24	30.0
No	56	70.0
Total	80	100

## Abbreviations

HNSCC: head and neck squamous cell carcinoma
EGFR: epidermal growth factor receptor
SCC: squamous cell carcinomas
TKI: tyrosine kinase inhibitors
EGF: epidermal growth factor
NSCLC: non-small-cell lung carcinoma
ATCC: American Type Culture Collection
HOK: Human oral keratinocytes
SACC: salivary adenoid cystic carcinoma
DMEM: Dulbecco's modified Eagle's medium
FBS: fetal bovine serum
IP: Immunoprecipitation
RT-PCR: Reverse Transcription-Polymerase Chain Reaction
IHC: immunohistochemistry
ECD: extracellular domain
CHX: cycloheximide
BRD4: bromodomain-containing protein 4
