# Supplementary material for: EREG-driven oncogenesis of Head and Neck Squamous Cell Carcinoma exhibits higher sensitivity to Erlotinib therapy
Source: Theranostics. 2020 Aug 25;10(23):10589–605. doi: 10.7150/thno.47176 (PMC7482801; doi:10.7150/thno.47176)

**Figure S1. (related to Figure 1)**

**(A)** Western blotting analysis of EREG, p-EGFR and EGFR in HOK cells and 5 HNSCC cell lines. **(B-C)** Patient survival data obtained from TCGA were analyzed based on the mRNA expression level of EREG. **(D)** Patient survival data obtained from TCGA were analyzed based on the mRNA expression level of EREG combined with EGFR. **(E-G**) High EREG expression significantly correlates with the poor survival rates of patients with different types of cancer, including cervical cancer, pancreatic cancer and lung cancer.

**Figure S2.** (**related to Figure 2)**

**(A-C)** Colony-formation analysis was performed on HN13 and CAL27 cell lines with or without epiregulin (50 ng/ml) treatment. The number of colonies was calculated. **(D)** Western blot analysis of EREG in HN4 cells stably expressing control vectors or EREG-specific shRNAs. **(E)** Western blot analysis of EREG in HN6 cells stably expressing control vectors or Flag-EREG.

**Figure S3. (related to Figure 3)**

**(A)** Immunoblot (IB) of HN13 and HN6 cells treated with epiregulin (50 ng/ml) at the indicated time points and probed with an anti-phosphotyrosine (p-Tyr) antibody. **(B)** The HEK293 GFP-EGFR cell line was transfected with HA-tag control or HA-EREG, and the intensity of GFP was examined under a fluorescence microscope. **(C)** Immunofluorescence staining for ErbB2, ErbB3 and Axl in HN6 cells treated with 50 ng/mL epiregulin at various time points.

**Figure S4. (related to Figure 5)**

**(A)** Endogenous EREG was immunoprecipitated from FaDu cells, and bound endogenous EGFR was examined by Western blotting. **(B)** HEK293 cells were transiently coexpressed with GFP-EREG and HA-tagged EGFR. The cellular location of EGFR (red) and EREG (green) was examined by immunofluorescence staining. Scale bar, 50 μm. **(C)** Inputs of Figure 4E and 4F.

**(D)** HEK293 cells were transiently coexpressed with GFP-tagged EREG and WT-EGFR or EGFR-ΔD. The cellular location of EGFR (red) and EREG (green) was examined by immunofluorescence staining. Scale bar, 100 μm. **(E)** Inputs of Figure 4I and 4J. **(F)** HEK293 cells were transiently coexpressed with EGFR and WT-EREG or EREG-N47Q. The cellular location of EGFR (red) and EREG (green) was examined by immunofluorescence staining. Scale bar, 100 μm. **(G)** Western blot analyses of p-EGFR and EGFR from HOK cells that were treated with condition medium as indicated.

**Figure S5. (related to Figure 6)**

**(A)** Immunofluorescence staining for C-Myc and F-actin in HN6 and CAL27 cells treated with or without 50 ng/mL epiregulin for 2 h. Scale bar = 100 μm. **(B)** CAL27, HN6 and HN13 cells were pretreated with or without actinomycin D (0.5 mg/ml) for 1 h followed by epiregulin stimulation for 2 h. C-Myc was analyzed by Western blotting. **(C)** Tumor cell lines were treated with 2.5 or 10 mM MG132 for 4 h. Endogenous C-Myc was examined by Western blotting. **(D)** HN6 and CAL27 cells were treated with or without EREG (50 ng/mL) for 2 h, followed by incubation with CHX (10 μM) for an extended period of time. C-Myc levels were determined by Western blot analysis. **(E)** Graphic representation of the densitometry results for C-Myc after CHX treatment (circle, with epiregulin; square, without epiregulin). **(F)** CAL27, HN6 and HN13 cells were pretreated with various BET BD inhibitors for 1 h followed by stimulation with epiregulin for 2 h. C-Myc expression was examined by Western blot analysis. **(G)** HN30 and HN4 cells were treated with various BET BD inhibitors for 24 h, and C-Myc expression was examined by Western blot analysis. **(H)** Colony-formation analysis was performed on HN4 cells with or without IBET-1 (5 μM) treatment. **(I)** The growth of 3D-cultured HN6 cells related to Figure 5M. Each data point represents the mean value and standard deviation of 3 replicate

wells. **(J)** HN30 and HN4 cells were treated with increasing concentrations of JQ1 and MS436, and C-Myc expression was analyzed by Western blotting. **(K)** CAL27 cells were pretreated with or without actinomycin D (0.5 mg/ml) for 1 h followed by epiregulin stimulation for 2 h. C-Myc, p-EGFR and EGFR were analyzed by Western blotting related to Figure 5F.

**Figure S6. (related to Figure 7)**

**(A)** The IC50 of erlotinib in 6 HNSCC cell lines. **(B)** Western blot analysis of EREG in HN30 cells stably expressing control vectors or EREG-specific shRNA.

# Figure S1

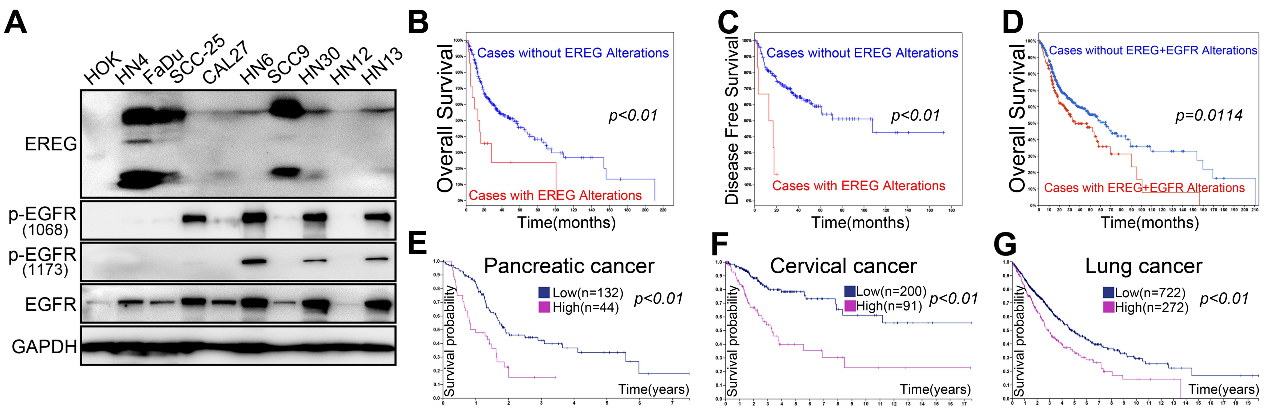
A
HOK
HN4
FaDu
SCC-25
CAL27
HN6
SCC9
HN30
HN12
HN13
EREG
p-EGFR (1068)
p-EGFR (1173)
EGFR
GAPDH
B
Overall Survival
Cases without EREG Alterations
p<0.01
Cases with EREG Alterations
Time(months)
C
Disease Free Survival
Cases without EREG Alterations
p<0.01
Cases with EREG Alterations
Time(months)
D
Overall Survival
Cases without EREG+EGFR Alterations
p=0.0114
Cases with EREG+EGFR Alterations
Time(months)
E
Pancreatic cancer
Low(n=132)
High(n=44)
p<0.01
Survival probability
Time(years)
F
Cervical cancer
Low(n=200)
High(n=91)
p<0.01
Survival probability
Time(years)
G
Lung cancer
Low(n=722)
High(n=272)
p<0.01
Survival probability
Time(years)

# Figure S2

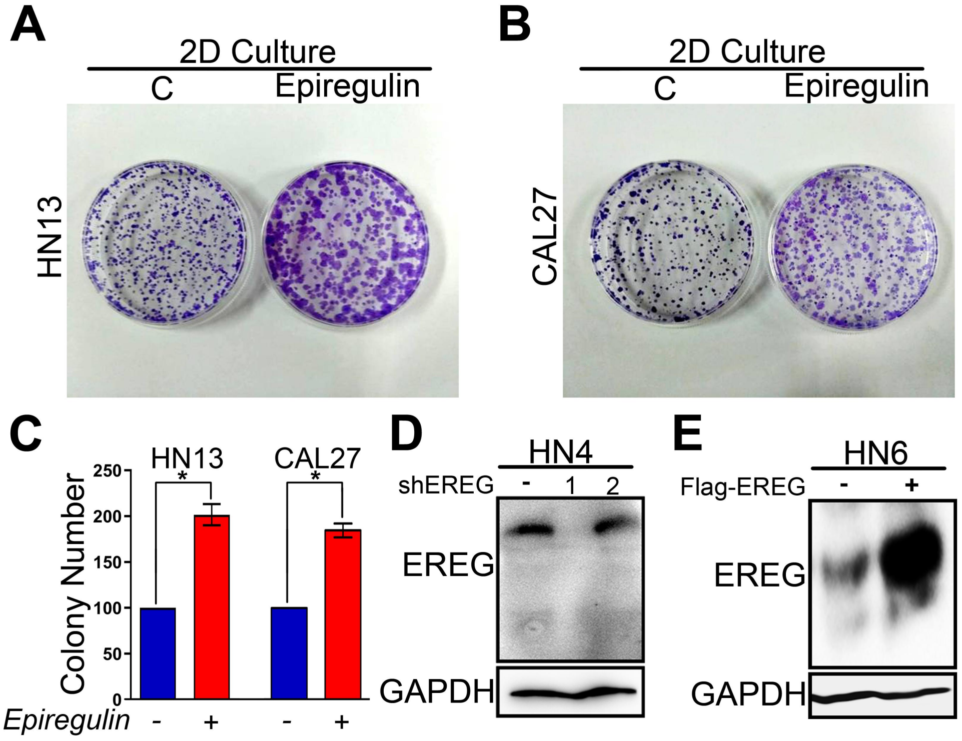
A
2D Culture
C
Epiregulin
HN13
B
2D Culture
C
Epiregulin
CAL27
C
HN13
CAL27
Colony Number
250
200
150
100
50
0
Epiregulin - + - +
D
HN4
shEREG - 1 2
EREG
GAPDH
E
HN6
Flag-EREG - +
EREG
GAPDH

# Figure S3

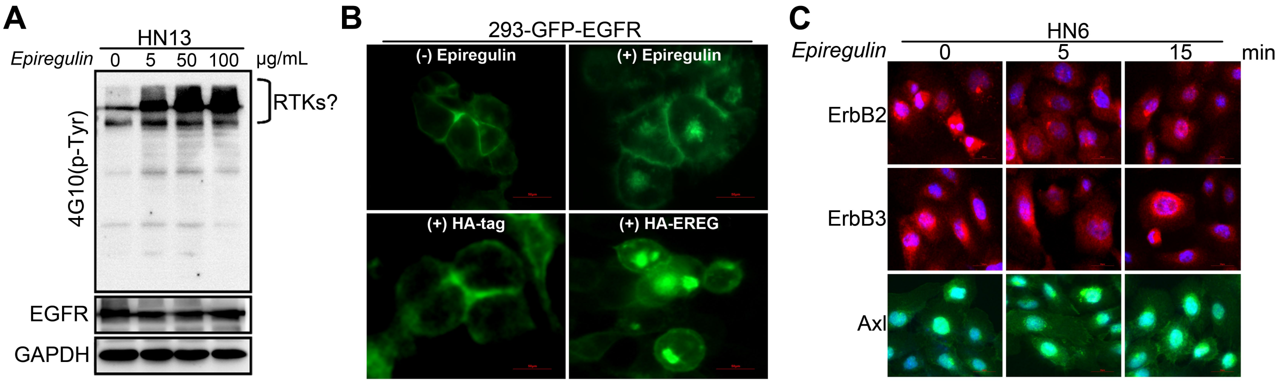

A
HN13
Epiregulin 0 5 50 100 μg/mL
4G10(p-Tyr)
RTKs?
EGFR
GAPDH
B
293-GFP-EGFR
(-) Epiregulin
(+) Epiregulin
(+) HA-tag
(+) HA-EREG
C
HN6
Epiregulin 0 5 15 min
ErbB2
ErbB3
Axl

# Figure S4

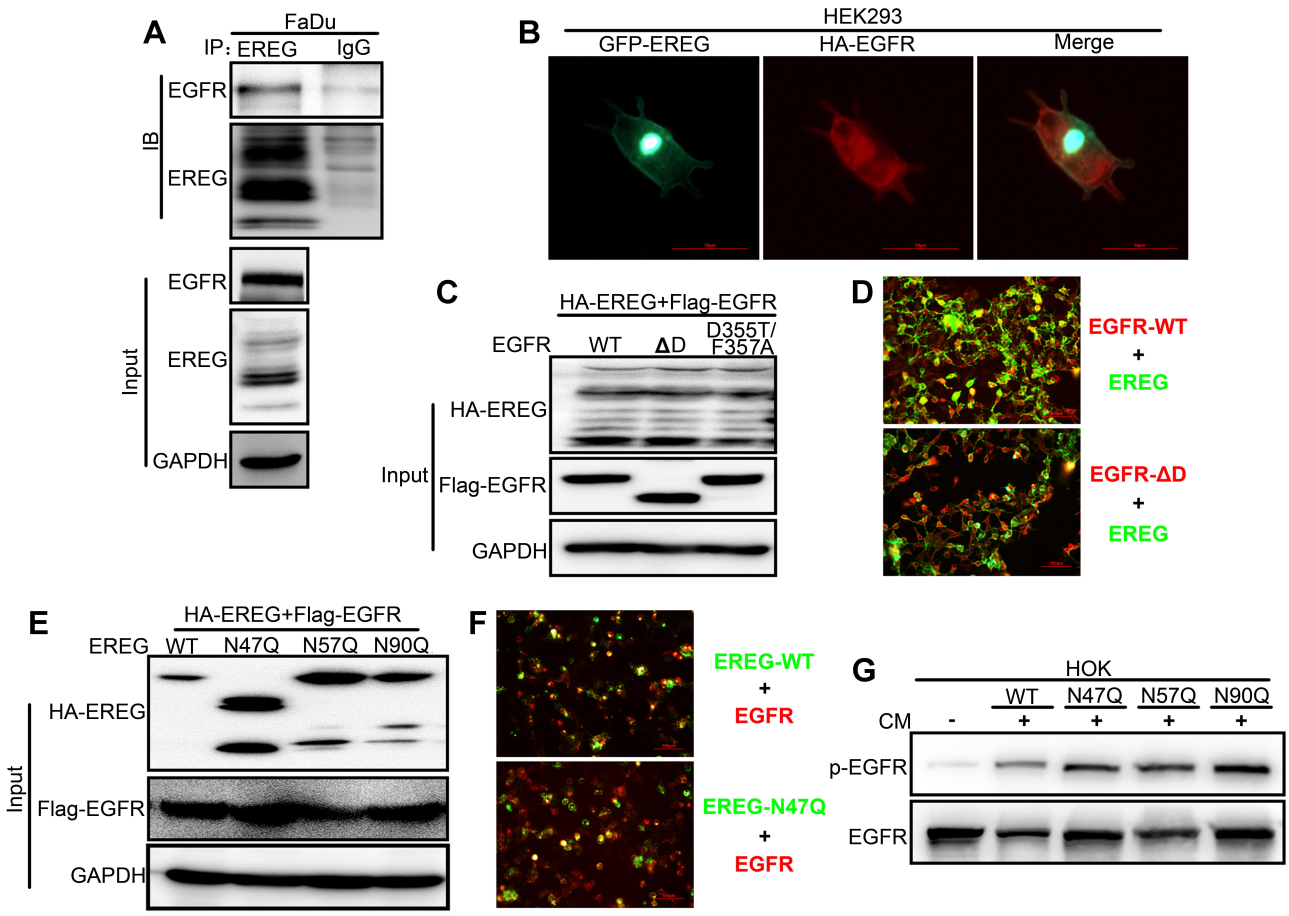
A
FaDu
IP: EREG
IgG
IB
EGFR
EREG
Input
EGFR
EREG
GAPDH
B
HEK293
GFP-EREG
HA-EGFR
Merge
C
HA-EREG+Flag-EGFR
EGFR
WT
ΔD
D355T/F357A
Input
HA-EREG
Flag-EGFR
GAPDH
D
EGFR-WT
+
EREG
EGFR-ΔD
+
EREG
E
HA-EREG+Flag-EGFR
EREG
WT
N47Q
N57Q
N90Q
Input
HA-EREG
Flag-EGFR
GAPDH
F
EREG-WT
+
EGFR
EREG-N47Q
+
EGFR
G
HOK
WT
N47Q
N57Q
N90Q
CM
-
+
+
+
+
p-EGFR
EGFR

# Figure S5

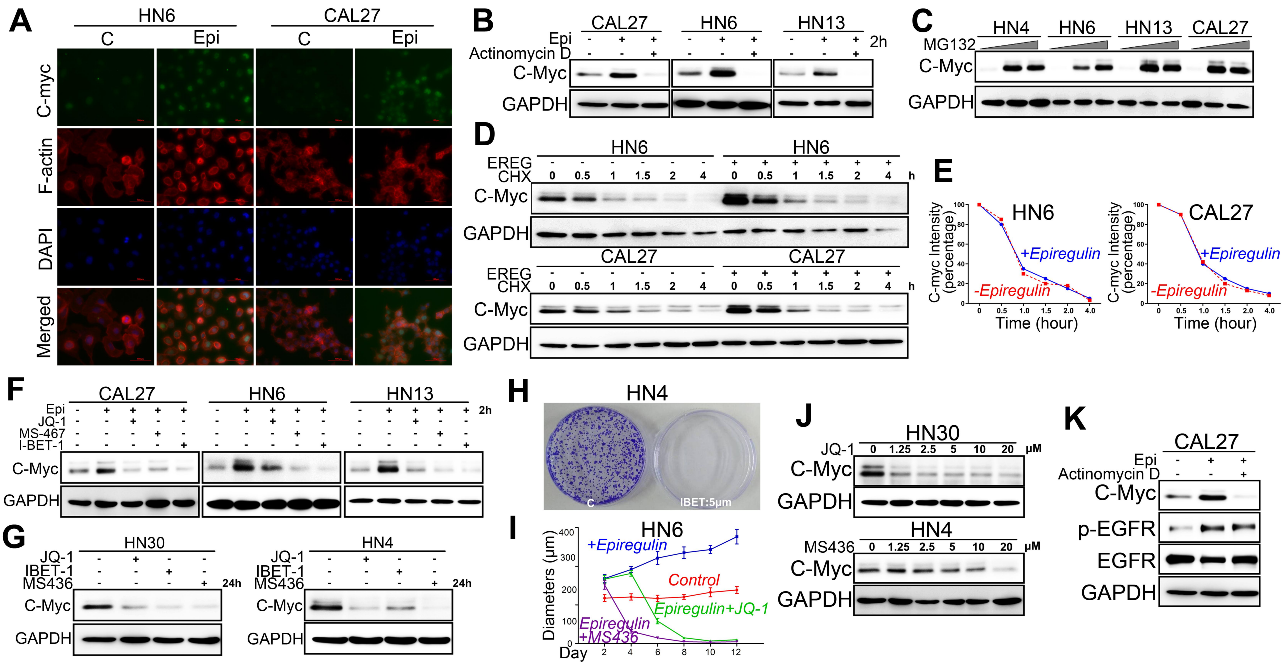

A
HN6
CAL27
C
Epi
C-myc
F-actin
DAPI
Merged
B
CAL27
HN6
HN13
Epi
Actinomycin D
2h
C-Myc
GAPDH
C
MG132
HN4
HN6
HN13
CAL27
C-Myc
GAPDH
D
HN6
EREG
CHX
0 0.5 1 1.5 2 4
h
C-Myc
GAPDH
CAL27
E
HN6
CAL27
C-myc Intensity (percentage)
+Epiregulin
-Epiregulin
Time (hour)
F
CAL27
HN6
HN13
Epi
JQ-1
MS-467
I-BET-1
2h
C-Myc
GAPDH
G
HN30
HN4
JQ-1
IBET-1
MS436
24h
C-Myc
GAPDH
H
HN4
C
IBET:5μm
I
HN6
Diameters (μm)
+Epiregulin
Control
Epiregulin+JQ-1
Epiregulin +MS436
Day
J
HN30
JQ-1
0 1.25 2.5 5 10 20
μM
C-Myc
GAPDH
HN4
MS436
K
CAL27
Epi
Actinomycin D
C-Myc
p-EGFR
EGFR
GAPDH

# Figure S6

A

| Cell lines | IC50 (μM) |
|---|---|
| HN30 | 4.6 |
| FaDu | 8.2 |
| HN4 | 8.4 |
| HN12 | 52.6 |
| CAL27 | 4.4 |
| SACC-83 | 24.8 |

B

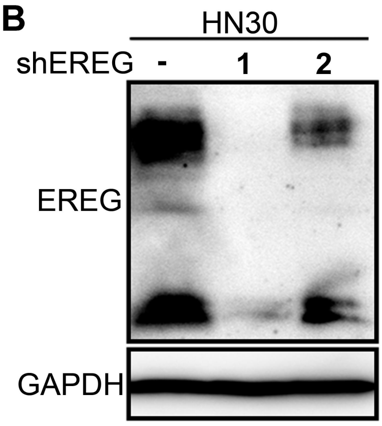

Supplement: Supplementary file 1 — Supplementary figures. [file thnov10p10589s1.pdf]
